# Closing the air gap: the use of drones for studying wildlife ecophysiology

**DOI:** 10.1111/brv.13181

**Published:** 2025-01-17

**Authors:** Adam Yaney‐Keller, Rebecca R. McIntosh, Rohan H. Clarke, Richard D. Reina

**Affiliations:** ^1^ School of Biological Sciences, Monash University 25 Rainforest Walk Clayton Victoria 3800 Australia; ^2^ Research Department Phillip Island Nature Parks 154/156 Thompson Avenue, Cowes Victoria 3922 Australia

**Keywords:** ecophysiology, unoccupied aerial vehicles, remotely piloted aircraft, unmanned aerial vehicles, remote sensing, body condition, morphometrics, kinematics, bioenergetics, wildlife health

## Abstract

Techniques for non‐invasive sampling of ecophysiological data in wild animals have been developed in response to challenges associated with studying captive animals or using invasive methods. Of these, drones, also known as Unoccupied Aerial Vehicles (UAVs), and their associated sensors, have emerged as a promising tool in the ecophysiology toolkit. In this review, we synthesise research in a scoping review on the use of drones for studying wildlife ecophysiology using the PRISMA‐SCr checklist and identify where efforts have been focused and where knowledge gaps remain. We use these results to explore current best practices and challenges and provide recommendations for future use.

In 136 studies published since 2010, drones aided studies on wild animal body condition and morphometrics, kinematics and biomechanics, bioenergetics, and wildlife health (e.g. microbiomes, endocrinology, and disease) in both aquatic and terrestrial environments. Focal taxa are biased towards marine mammals, particularly cetaceans. While conducted globally, research is primarily led by institutions based in North America, Oceania, and Europe. The use of drones to obtain body condition and morphometric data through standard colour sensors and single camera photogrammetry predominates. Techniques such as video tracking and thermal imaging have also allowed insights into other aspects of wildlife ecophysiology, particularly when combined with external sampling techniques such as biologgers. While most studies have used commercially available multirotor platforms and standard colour sensors, the modification of drones to collect samples, and integration with external sampling techniques, have allowed multidisciplinary studies to integrate a suite of remote sensing methods more fully.

We outline how technological advances for drones will play a key role in the delivery of both novel and improved wildlife ecophysiological data. We recommend that researchers prepare for the influx of drone‐assisted advancements in wildlife ecophysiology through multidisciplinary and cross‐institutional collaborations. We describe best practices to diversify across species and environments and use current data sources and technologies for more comprehensive results.

## I. INTRODUCTION

Physiological studies of free‐ranging wildlife are critical for answering the fundamental biological questions of *how* and *why* animals interact with each other and their environment (Wikelski & Cooke, 2006). Further, these answers are needed in order to address modern conservation challenges (White *et al*., 2007). For example, the impact of pesticides on songbirds highlighted in Rachel Carson's *Silent Spring* (Carson, 1962) emphasises the role of physiological research in shaping environmental regulations (Pollock, 2001). Building on this, contemporary studies that seek to understand how habitat fragmentation (e.g. Johnstone, Reina & Lill, 2012) or pollutants (e.g. Wikelski *et al*., 2002) increase stress levels in free‐ranging wildlife can show how these effects may influence recruitment and recovery in threatened populations.

The collection of physiological data has often involved measurements from animals in permanent or temporary captivity, both of which may produce different results when compared with fully wild counterparts (Turko *et al*., 2023). If fully wild sampling does occur, studies may utilise invasive sampling techniques, such as capture, restraint and surgical or temporary attachment of biologging devices to the body. These approaches may negatively impact wildlife behaviour or physiology, potentially confound the data being collected (Johnstone *et al*., 2012), pose potential risks to both the animals and researchers involved, and may require significant financial investment. This has led to increased calls for exploration of more non‐invasive sampling methods that leverage advances in technology to maximise researcher safety and capability, reduce impacts on animals, and increase data accuracy (Zemanova, 2020; Fahlman *et al*., 2021). In response, several non‐invasive techniques, often adapted from innovations in fields such as human health and remote sensing, have been pioneered in recent decades (Hawkes, Fahlman & Sato, 2021; MacDonald, Hawkes & Corrigan, 2021; Williams *et al*., 2021). This has included reductions in the size and impact of biologging devices (e.g. Aoki *et al*., 2021b; Fahlman *et al*., 2021), analysis of hormones or DNA from hair and feathers or the environment (e.g. Waits & Paetkau, 2005; Babic *et al*., 2022; Schilling, Mazzamuto & Romeo, 2022) and the use of drones, also known as Unoccupied Aerial Vehicles (UAVs) or Remotely Piloted Aircraft Systems (RPAS) (Linchant *et al*., 2015).

In recent decades, drones have played a significant role in advancing the field of wildlife ecology (Koh & Wich, 2012; Hodgson *et al*., 2016). Their relatively low cost, ease of use, rapid deployment capabilities, and increased safety compared to traditional aerial surveys have led to widespread adoption as a tool for free‐ranging wildlife research (Schofield *et al*., 2017; Sorrell *et al*., 2019; Yaney‐Keller, San Martin & Reina, 2021; Weinstein *et al*., 2022). Reviews on the use of drones across disciplines, ecosystems, and taxa (e.g. Rees *et al*., 2018; Jiménez López & Mulero‐Pázmány, 2019; Hyun, Park & Lee, 2020; Oleksyn *et al*., 2021; Schad & Fischer, 2023; Tuia *et al*., 2022; Álvarez‐González *et al*., 2023) and best practices for data collection, meeting legislative requirements, and reporting protocols (e.g. Junda, Greene & Bird, 2015; Brack, Kindel & Oliveira, 2018; Duffy *et al*., 2018; Barnas *et al*., 2020; Raoult *et al*., 2020; Sorrell *et al*., 2023) are relatively common within contemporary scientific literature. Nevertheless, the use of drones for studying wildlife physiology and ecophysiology (hereafter used to encompass both terms) has not yet received the same focus (Chabot & Bird, 2015; Mo & Bonatakis, 2022).

Common and cost‐effective off‐the‐shelf drones often come with an in‐built colour sensor, potentially biasing applications in wildlife biology and ecology to research where taxa can be easily quantified using colour imagery (Linchant *et al*., 2015). A recent review of research on wildlife using drones by Mo & Bonatakis (2022) found that population and demographic surveys, animal detection, and investigations of animal responses to drone flights, collectively made up 84% of published literature in the field. This apparent preference may be a consequence of (*i*) the novelty of the tool and the need to validate its use; (*ii*) inherent difficulty in measuring a physical attribute remotely; (*iii*) the comparative ease of applying a new tool to established methods rather than developing and validating new techniques; and (*iv*) a need to maintain adequate distance and flight parameters to fulfil regulatory guidelines and minimise disturbance or other risks to study animals (Chabot & Bird, 2015; McIntosh, Holmberg & Dann, 2018; Raoult *et al*., 2020).

The need to collect ecophysiological data from free‐ranging wildlife, the utility and ethical priority of researchers to sample such parameters non‐invasively where possible, and the technological revolution of drones has further lifted innovation in this field. With improved data collection capabilities at reduced cost, drones are increasingly accessible for wildlife research, resulting in a wider adoption for wildlife ecophysiology studies. Here, we conduct a scoping review on the application of drones to the study of wildlife ecophysiology. Specifically, we seek to (*i*) quantify where research efforts have been focused to date; (*ii*) understand current research trends within the field and explore this to highlight knowledge gaps; and (*iii*) provide an overview of current best practices, challenges, and recommendations to guide future usage.

## II. METHODS

### (1) Literature search

This review follows guidelines established in the Preferred Reporting Items for Systematic review and Meta‐Analysis extension for scoping reviews (PRISMA‐ScR) checklist (Tricco *et al*., 2018). Our study‐specific PRISMA flow diagram is presented in the online Supporting Information (Fig. S1).

Published studies were identified for inclusion by searching *Web of Science*™ and *Google Scholar* in two searches conducted in January 2024 and July 2024. See Table S1 for full list of search terms, filters, and search dates. The *Web of Science* search used the formula “Topic = (drone OR drones OR remotely piloted aircraft OR unmanned aerial vehicle OR unmanned aerial system OR unpiloted aircraft system OR unoccupied aerial system) AND All Fields = (physiology OR ecophysiology OR health OR stress OR injury OR disease OR body condition OR morphometrics OR biomechanics OR kinematics OR energy OR thermoregulation OR metabolic rate OR bioenergetics OR growth OR respiration OR heart rate OR thermal) AND All Fields = (wildlife OR animals OR vertebrate OR invertebrate OR terrestrial OR marine OR freshwater OR coastal)”. *Web of Science* results were filtered to exclude review articles, search terms indicating studies involving either European honeybees (*Apis mellifera*, where ‘drones’ is a homonym), common laboratory experimental species not likely to be studied by a drone (i.e. *Drosophila*, zebrafish), and studies that were not categorised as ecophysiology per *Web of Science* categories (assigned by Clarivate at the journal level) (Table S1). The *Google Scholar* search was conducted in Publish or Perish software (Harzing, 2007) with a maximum limit of 1000 studies using the formula (Drone OR “remotely piloted aircraft” OR “unmanned aerial vehicle” OR “unpiloted aircraft system” OR “unoccupied aerial system” AND physiology OR ecophysiology OR health OR stress OR injury OR disease OR body condition OR biomechanics OR kinematics OR energy OR thermoregulation AND wildlife). Numerical results from each search iteration and filtering process are provided in Fig. S1.

### (2) Articles screening and classification

Search results from *Web of Science* and *Google Scholar* were formatted using EndNote X9 (The EndNote Team, 2013) and uploaded into the program Covidence (Veritas Health Innovation, 2024). Covidence was used to remove duplicates after which a single reviewer (A. Y.‐K.) conducted an initial screening by title and abstract followed by a full‐text screening using the inclusion criteria listed in Table 1. The number of duplicates as well as the included and excluded results are presented in Fig. S1.

**Table 1:** Inclusion and exclusion criteria for studies to be included in the scoping review.

Criteria	
***Inclusion*** *(must meet all criteria for inclusion)*	The study was published before January 01, 2024, including studies published online in 2023 but appearing in a 2024 volume.
	The study was published in English.
	The study was a peer‐reviewed article, note or brief communication, or case study.
	The study reported wildlife physiological metrics obtained *via* a drone platform (e.g. heart rate, temperature, morphometrics) even if the initial purpose of the study did not focus specifically on wildlife ecophysiology.
	The study used products produced by a drone platform to infer, inform, or calculate metrics relevant to ecophysiology (e.g. body condition, oxygen consumptive rate, speed of movement), alone or in conjunction with other (i.e. non‐drone based) methods.
***Exclusion*** *(any single criterion qualifies for exclusion)*	The study was a review article.
	The study was grey literature (e.g. conference proceedings and papers, pre‐prints, theses, working papers).
	The study involved only fully domesticated species (species that may be considered feral or not domesticated but housed and/or bred in captivity were retained; Purugganan, 2022).

### (3) Database construction

From each included paper, we extracted information on the relevant ecophysiological topic(s) that was explored as defined by six *a priori* research topic categories (Table 2). Information was also extracted on the (*i*) environment in which the drone collected data (marine, freshwater, coastal, or inland), (*ii*) study species and taxonomic groupings, (*iii*) research location (country and continent), (*iv*) location of the lead author's institution (country and continent), (*v*) drone(s) used [multirotor (commercial or modified/custom) and/or fixed‐wing (commercial or modified/custom)], make, model, and weight class (micro, very small, small, medium, or large), (*vi*) drone‐borne sensor(s) and hardware used for data collection, (*vii*) non‐drone‐borne sensor(s) and hardware used for data collection, (*viii*) data‐processing techniques and product(s) created, (*ix*) metrics reported and whether they came from drone‐ or non‐drone‐borne means, (*x*) metrics relevant to ecophysiology calculated or inferred from data collected, and (*xi*) calibration or correction method applied (if applicable). See Table S2 for further explanation of each parameter collected and Data S1 for the full data set of extracted information collected from each study included in this review.

**Table 2:** Research topics within ecophysiology which have been studied using drones, as covered in this review.

Research topic	Description
*Body Condition and Morphometrics*	Measurement of outward physical characteristics (e.g. size, girth, mass) to derive anatomical measurements or indices of body condition.
*Kinematics and Biomechanics*	Measurement of physical motion or the structure, function, or mechanical aspects of animal movement. Excludes tracking animal movement through space or interactions with other individuals (i.e. focuses on how animals move, not the purpose of that movement).
*Bioenergetics*	Measurement of acquisition and allocation of energy to support maintenance, activity, growth and reproduction.
*Vital Signs: Respiration, Heart Rate, and Temperature*	Measurement of respiration, heart rates and body or surface temperature, including physiological aspects of each.
*Microbiome*, *Endocrinology, and Genetics*	Sampling and analysis of biological materials to determine attributes of animal microbiome and/or virome, endocrine function, and/or genetics.
*Disease and Injury*	Determination of the effect or prevalence of disease and/or injury of a study animal or population.

Each ecophysiology topic is organised into the following sections: (*i*) background, which provides context and a brief discussion of relevant methods that employ drones; (*ii*) trends and implications; which focuses on identifying emerging patterns, directions in research, and major areas of exploration, as well as an assessment of where research efforts have been less explored and are yet outstanding; and (*iii*) challenges and opportunities, which examines the key difficulties or barriers encountered in the use of drones to date and explores potential opportunities for innovation, improvement, and future research directions.

## III. GENERAL TRENDS

**Fig. 1:**
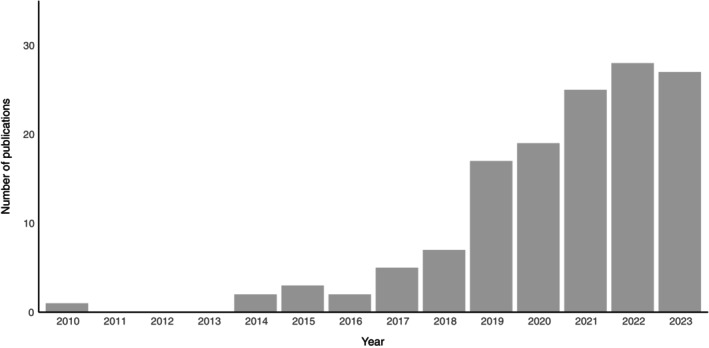
Number of included publications involving the use of drones for studying wildlife ecophysiology per year.

Using the specified criteria (Table 1) we identified 136 studies that utilised drones for the study of wildlife ecophysiology. There was a trend for an increasing number of studies over time, with a single ecophysiological study published in 2010 and 27 studies published in 2023 (Fig. 1). Studies were heavily biased to aquatic environments (*N* = 100 marine, *N* = 7 freshwater), when compared with terrestrial environments (*N* = 12 coastal, *N* = 16 inland). Studies typically involved mammals (*N* = 112), with fish (*N* = 13), reptiles (*N* = 10), birds (*N* = 1), and jellyfish (*N* = 1) also represented (Table S3). Within mammals, most studies focused on marine mammals (*N* = 100), particularly cetaceans (*N* = 86), but also pinnipeds (*N* = 11). Other animal groups included chondrichthyans [sharks (*N* = 5) and rays (*N* = 4)], ungulates (*N* = 8), crocodilians (*N* = 5), primates (*N* = 3), and lizards, sea turtles, and bony fish (*N* = 2 each), as well as individual species from other vertebrate and invertebrate groups. In total the ecophysiology of 63 species has been studied using drones (Table S3).

The bias towards the study of the marine environment and its associated megafauna may stem from several factors. The marine environment is comparatively obstacle‐free and uncluttered visually compared to the terrestrial environment (e.g. lack of obscuring vegetation). Additionally, as water is often transparent to a considerable depth, an aerial perspective allows for observation of attributes or behaviours that are below the water's surface which may not be seen from the lower angles of observation from vessel or land‐based approaches (Fiori *et al*., 2017). Further, fully aquatic animals are generally less reactive to drones than terrestrial species (Mulero‐Pázmány *et al*., 2017). This may be due to a higher sensitivity in terrestrial animals to visual and auditory cues from drones, which provide corollaries with aerial predation or swarming bees, issues not experienced by many large marine taxa (Bennitt *et al*., 2019; Raoult *et al*., 2020). The perceived sound of drones is also higher above water than below, decreases with depth, and may be masked further by ambient conditions, adding to their relative convenience of use for marine animals (Christiansen *et al*., 2016b; Erbe *et al*., 2017; Laute *et al*., 2023). For these reasons, drones may be particularly advantageous for studying near‐surface marine animals, offering lower risks and greater benefits compared to vessel‐, snorkel‐, or scuba‐based approaches. Conversely, for terrestrial animals, drones may be relatively less convenient than approaching on foot or by vehicle if the degree of visual obstruction and/or risk of disturbance from the drone is high. Moreover, the employment of novel technologies is often driven by need. Direct measurement, although often invasive, and at a minimum requiring capture and restraint, can yield data rapidly and accurately whereas remote measurement may only provide estimates with relative confidence after intensive calibration and validation (McCafferty, Gallon & Nord, 2015; Palme, 2019; Williams *et al*., 2021). Terrestrial animals are typically easier to capture, restrain, and measure for fundamental ecophysiological metrics (e.g. body mass, fat content, body temperature) than marine animals, and many measurement methods have been validated over decades. Marine animal ecophysiology has historically been studied in captivity, which is infeasible for large cetaceans and many other marine species. Researchers studying marine wildlife, particularly marine mammals, need effective tools for studying hard‐to‐capture, cryptic, and often wide‐ranging species, and thus have adopted these techniques rapidly and expanded the use of drones in their research, including in ecophysiology.

**Fig. 2:**
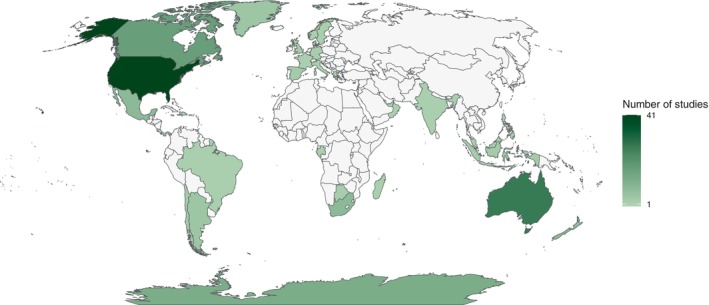
Countries and the continent of Antarctica containing study sites where drones were used to study wildlife ecophysiology.

**Fig. 3:**
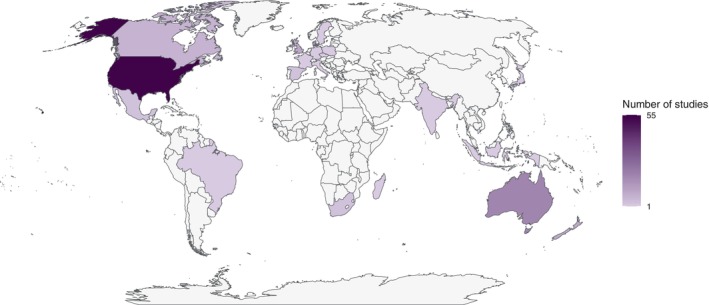
Countries of institutions of lead authors of publications using drones for studying wildlife ecophysiology.

Studies were primarily conducted in North America (*N* = 62) and Oceania (*N* = 36), with studies also undertaken in Central America and the Caribbean (combined = 15), Europe and Africa (*N* = 15 each), Antarctica (*N* = 12), Asia (*N* = 11), and South America (*N* = 9) (Fig. 2). The institution of the lead author was most often located in North America (*N* = 60), Europe (*N* = 33), and Oceania (*N* = 28) (Fig. 3). There was a geographic bias towards more developed nations and their respective waters hosting more study sites [e.g. U.S.A. (*N* = 41), Australia (*N* = 27), Canada (*N* = 17)] and lead author institutions [e.g. U.S.A. (*N* = 55), Australia (*N* = 19), Denmark (*N* = 15)]. However, there was a wider geographic spread in study sites than in the locations of lead author institutions (Figs 2 and 3). This suggests the global application of drones in ecophysiological studies is in part supported by investigators from institutions in more developed nations. The reasons for this are complex but may be influenced by availability of funding, access to equipment, and opportunities for mutually beneficial collaborations between visiting and host nation researchers.

We found that drones have primarily been used to obtain body condition and morphometric data (*N* = 101). All studies used very small (250 g – 2 kg, *N* = 116) and/or small (2–25 kg, *N* = 88) weight class drones. The type of drone used most often was an unmodified, commercial multirotor platform (*N* = 93), with only six studies utilising a fixed‐wing drone platform. However, custom or modified multirotor systems (*N* = 46) (e.g. with sample plates attached to the drone, *N* = 8), or those paired with other external equipment or sampling techniques (e.g. biologgers, *N* = 8), have been applied simultaneously to address several ecophysiological topics (Table S3).

## IV. BODY CONDITION AND MORPHOMETRICS

### (1) Background

Animal morphology (e.g. size, shape, and mass) influences life history, fitness, and foraging capabilities, and is fundamental to understanding physiology and ecology (Kleiber, 1975). Body condition, the relative energy reserves of an animal given its structural size, is often measured by morphological indices created by some combination of body width, mass and/or linear dimensions, such as ratios (e.g. body mass divided by length) and residuals (e.g. derived from a regression of mass on length) (Hayes & Shonkwiler, 2001; Stevenson & Woods Jr, 2006). The choice of index varies, as different species may allocate resources to different areas of the body (Hayes & Shonkwiler, 2001). The calculation of a body condition index *via* drone imagery is discussed here rather than bioenergetics (see Section VI), despite it being informative for this latter topic, because the calculation of such indices from remote imagery requires the measurement of at least one anatomical feature. Obtaining this index *via* drone imagery has the added benefit of being non‐invasive compared to physically measuring an animal.

Aerial photogrammetry is the science of obtaining reliable measurements from aerial photographs (McGlone, 2013) and has been used by wildlife researchers to derive measurements of large, difficult‐to‐capture animals for nearly half a century (Croze, 1972; Whitehead & Payne, 1982). Most drone‐based morphometric studies (*N* = 88) use single camera photogrammetry (SCP), where a camera mounted on a drone captures full‐body images of target animals. Using the known height of the drone, pixel dimensions of the viewing window, focal length and size of the sensor, the ground sampling distance (GSD) can be calculated, a metric of how much real‐world distance or area in the photograph each pixel represents (McGlone, 2013). This technique is often used to measure two‐dimensional (2D) attributes such as body length, width, or dorsal surface area (Goebel *et al*., 2015; Christiansen *et al*., 2016a). Three‐dimensional (3D) measurements, like body volume (Christiansen *et al*., 2018), can also be estimated by modelling from 2D measurements such as body height (Christiansen *et al*., 2019). Wildlife morphometrics can also be obtained using Structure‐from‐Motion (SfM) drone photogrammetry (*N* = 22), a technique that uses overlapping drone‐derived photographs from a moving camera to model a point cloud and mesh from which 2D orthomosaics or 3D model products can be produced (Ullman, 1979). Similar 2D [e.g. length and surface area (Ezat, Fritsch & Downs, 2018; Raoult & Gaston, 2018)] or 3D (e.g. body volume; Hodgson *et al*., 2020) morphometrics can then be measured based on the GSD of those products from direct georeferencing. Although drone‐SfM, like SCP, is often performed with a single camera, it relies on overlapping photographs from a moving camera to estimate point positions from varying perspectives, while SCP measures a feature in a single image frame (Ullman, 1979; Whitehead & Payne, 1982; Mathews *et al*., 2023). We treat SfM and SCP as distinct techniques in this review. From both SCP and SfM, morphometrics alone may be useful for determining age or sex classifications for certain species (Christiansen *et al*., 2016a; Fortune *et al*., 2017), and health‐related metrics such as body condition and body mass can be obtained with further calculations (Christiansen *et al*., 2016a; Krause *et al*., 2017; Raoult & Gaston, 2018; Allan *et al*., 2019).

### (2) Trends and implications

A total of 77% (*N* = 101) of studies explored morphometric derivation using drone platforms and marine mammal morphometrics constituted a substantial portion of this, accounting for about 60% (*N* = 79) of all publications. This focus is particularly relevant given the important role of lipid storage in blubber as a primary energy reserve for cetaceans and pinnipeds, with changes in blubber mass leading to large changes in body size reflective of overall health (Brodie, 1975; Ryg, Smith & Øritsland, 1988; Costa & Williams, 1999). As such, using drones to estimate mass, ratios of length and width, and body volume have been invaluable to assess morphometrics and body condition in free‐ranging marine mammals (Pomeroy, O'Connor & Davies, 2015; Christiansen *et al*., 2016a, 2018; Krause *et al*., 2017). The field has progressed most rapidly in cetacean research, where obtaining morphometric measurements was traditionally limited to dead or stranded individuals (Read, 1990; Víkingsson, 1995; Stevick, 1999). Indeed, drone photogrammetry has revolutionised this practice, enabling non‐invasive body measurements of diverse cetacean species across all oceans (Table S3).

Morphometric measurements derived from SCP have been used to examine body size in both small (de Oliveira *et al*., 2023) and large (Durban *et al*., 2015b) cetaceans, enabling comparison and classification of ecotypes and subspecies (e.g. Leslie *et al*., 2020; Durban *et al*., 2021; Kotik *et al*., 2023). Calculation and comparison of body condition indices has allowed researchers to detect changes in body condition in different breeding and foraging grounds (e.g. Nielsen *et al*., 2019; Fearnbach *et al*., 2020; Lemos *et al*., 2020a; Aoki *et al*., 2021a; Barlow *et al*., 2023), understand the energetic costs of gestation, lactation, and migration (e.g. Christiansen *et al*., 2016a, 2018, 2020b; Russell *et al*., 2022), and examine the effects of anthropogenic stressors on individual body condition (e.g. Christiansen *et al*., 2020a; Stewart *et al*., 2021b; Lemos *et al*., 2022a; Charlton *et al*., 2023; Pirotta *et al*., 2023). Combining body size metrics and subsequent condition indices with those gained from long‐term data sets can also yield powerful information on population‐level health through time and space (e.g. Groskreutz *et al*., 2019; Stewart *et al*., 2021a, 2023; Torres *et al*., 2022; Vermeulen *et al*., 2023). Moreover, multidisciplinary studies have integrated drone‐derived morphometrics with biologging and external sampling to offer insights into kinematics, bioenergetics, and endocrinology in, for example, baleen whales (e.g. Gough *et al*., 2021, 2022; Lemos *et al*., 2022b; Fernandez Ajó *et al*., 2023).

In pinnipeds, researchers have used either SCP (Goebel *et al*., 2015; Pomeroy *et al*., 2015; Krause *et al*., 2017; Alvarado *et al*., 2020), or SfM‐derived orthomosaics (Allan *et al*., 2019; Fudala & Bialik, 2020; Hodgson *et al*., 2020) to capture morphometrics including length, width, and body area to interpret body condition in individual seals ashore. At scale, morphometrics measured from SfM products can be used to gain information on colony‐wide breeding dynamics through size‐based age classifications (Fudala & Bialik, 2020) or 3D volumetrics or automated measurement workflows can be integrated to expand body condition measurements to groups or populations (Shero *et al*., 2021; Infantes *et al*., 2022).

Although less extensive than in marine mammals, ecophysiological research on other marine animals has employed both SCP and SfM‐derived morphometrics to assess size and body condition in sharks, rays, manatees, tuna, sea turtles, and jellyfish (Table S3). For example, morphometrics measured from drones have been used to examine how body size influences white shark (*Carcharodon carcharias*) movement in relation to habitat types and foraging opportunities (Colefax *et al*., 2020; Tucker *et al*., 2021), differentiate immature sea turtles of different species occupying a high‐density foraging habitat (Stokes *et al*., 2023), and provide information on sexual dimorphism, growth rates, habitat usage and nursery locations of reef manta rays (*Manta alfredi*) (Setyawan *et al*., 2022a,b).

While much of the focus in drone‐based morphometric studies has been on marine or coastal wildlife, several key studies have extended this approach to terrestrial and freshwater wildlife (Table S3). For instance, SfM‐derived body length and width measurements were useful for determining sex and pregnancy status, although not body mass, in Arabian oryx (*Oryx leucoryx*) (de Kock *et al*., 2021). Similarly, in Sumatran elephants (*Elephas maximus sumatrensis*), SfM‐derived body dimensions, validated by direct measurements of tagged individuals and analysed with machine learning, have been applied to assess body condition and age structure within an endangered population (Rahman *et al*., 2023). In freshwater environments, measurements of crocodilians have been derived from both SCP and SfM‐orthomosaic techniques (e.g. Ezat *et al*., 2018; Viljoen *et al*., 2023a); although incorporation of more robust calibration and validation methods in future studies would be of benefit. For example, Aubert *et al*. (2024) presented a comprehensive protocol for predicting total length from head length in diverse crocodilian species in drone images of submerged individuals, and included robust estimates of error and discussions of measurement uncertainty. In addition, measurements of body length and width in common hippopotamus (*Hippopotamus amphibius*) have been used to derive age structure and assess body condition in wild populations, accompanied by statistical techniques for extrapolating measures for submerged animals (Inman *et al*., 2019; Inman & Leggett, 2022). The development of approaches to obtain morphometrics from submerged animals highlights some of the challenges faced in obtaining these data with drones, such as individuals being partially obscured in turbid water, and the need to validate measurements and provide estimates of uncertainty.

### (3) Challenges and opportunities

Accurate drone‐based morphometric measurements depend on several factors that influence the precision and reliability of results. These include animal position and behaviour during photography (e.g. body flexion or movement) and environmental factors such as glare or vegetation obstructions (Bierlich *et al*., 2021a). To enhance measurement accuracy, researchers should score images based on quality indicators such as camera focus, body posture, and desired attribute measurability, using repeat assessments from multiple reviewers to ensure consistency (Cubbage & Calambokidis, 1987; Perryman & Lynn, 2002; Christiansen *et al*., 2016a, 2018). Furthermore, calculating coefficients of variation across single or multiple images of the same individual (Christiansen *et al*., 2016a; Burnett *et al*., 2019) and employing Bayesian statistical models to account better for uncertainty (Bierlich *et al*., 2021a,b) can improve precision and provide estimates of uncertainty in morphometric measurements. Using software that integrates such statistical approaches can further streamline the process (Torres & Bierlich, 2020; Bird & Bierlich, 2020). The use of barometric altimeters with real‐time temperature correction (Durban *et al*., 2015a; Goebel *et al*., 2015) or laser altimeters (LiDAR) (Dawson *et al*., 2017; Bierlich *et al*., 2023b), can help overcome many of the limitations of common barometric systems, and improve measured altitude precision and accuracy estimates for SCP (Sabatini & Genovese, 2013; Bierlich *et al*., 2021a). While LiDAR is less critical for deriving terrestrial animal morphometrics from SfM in open environments, it can improve height measurements in forested areas (Liao, Zhou & Yang, 2021; Rahman *et al*., 2023). Performing quality assessments and following best practice guidelines for SfM product creation is imperative. This includes the use of survey‐grade ground control and check points to help ensure spatial precision (Guan *et al*., 2022; Mathews *et al*., 2023). In addition, the use of real‐time‐kinematic or post‐processing kinematic satellite system‐enabled drones to improve GPS (global positioning system) precision may be valuable (Tomaštík *et al*., 2019; Famiglietti *et al*., 2021).

Body condition calculations are often robust to error caused by improper measurement of altitude, because relative body condition indices that are standardised against a metric of structural size (e.g. a ratio of body length to body width) should not be sensitive to altimeter error (Christiansen *et al*., 2016a; Burnett *et al*., 2019; Bierlich *et al*., 2021a). By contrast, absolute measures (e.g. total length) are often used to classify both aquatic and terrestrial animals into demographic units or determine the impacts of stressors on growth (Von Bertalanffy, 1938; Dmitriew, 2011; Eaton & Link, 2011). In these latter circumstances, altimeter error and thus measurement uncertainty are important considerations when inferring complex correlations between morphometrics and other life‐history parameters (Bierlich *et al*., 2021a).

Overall, the use of calibration and correction methods is encouragingly robust across studies measuring body condition and morphometrics from drone imagery (*N* = 104). This likely reflects the well‐developed methods for determining the morphometrics of marine and coastal species. Future studies aiming to use SCP or SfM drone photogrammetry to generate morphometrics and subsequent body condition indices in terrestrial environments, where the field remains in its infancy, should look to adapt the established methods used for marine mammals. Beyond this, investigators should aim to undertake and report research in line with best practice guidelines and protocols that have been established for drone photogrammetry and remote sensing, as well as wildlife research (James *et al*., 2019; Barnas *et al*., 2020; Mathews *et al*., 2023).

Another approach to drone photogrammetry that may overcome some of the limitations of SCP and SfM is the use of stereo‐video cameras (SVCs). This technique uses two or more cameras that occupy precisely different 3D space whilst recording the same object in order to determine its size through trigonometry, independent of altimeter accuracy and adaptable across various zoom configurations (Siegfried *et al*., 2021; Piacenza *et al*., 2022). SVC has been validated for a range of marine species using handheld devices, and a proof of concept system mounted on a commercially available multirotor drone has provided suitably accurate length measures of green sea turtles (*Chelonia mydas*) and nurse sharks (*Ginglymostoma cirratum*) (Piacenza *et al*., 2022). While current iterations of this system are in their infancy, future research using drone‐borne SVCs holds promise, particularly for smaller‐bodied focal species, where measurement errors may represent a significant proportion of total body size.

## V. KINEMATICS AND BIOMECHANICS

### (1) Background

The study of organismal movement and form has relevance for the study of evolution, feeding ecology, migration, and bioenergetics, all of which have conservation implications (Domenici & Seebacher, 2020). Until the advent of biologging, such studies were largely restricted to animals in temporary or permanent captivity. With animal‐borne GPS and inertial measurement units (IMUs) incorporating accelerometers, magnetometers, and gyrometers these tools can now capture fine‐scale movement for free‐ranging wildlife (Hawkes *et al*., 2021; Shield *et al*., 2023). However, the attachment of such biologgers typically involves significant disturbance to the study animal, often requiring physical or chemical immobilisation.

Remote measurement of kinematics and biomechanics in free‐ranging wildlife frequently relies on video tracking and analysis. For simple horizontal movement metrics like speed, two‐dimensional tracking of an animal's location can be achieved by comparing the visual distance travelled over time against objects of known size or position (Harvey *et al*., 2016; Basu *et al*., 2019; Werth *et al*., 2019), or using the drone's on‐board GPS if position is maintained above the target animal (Weir *et al*., 2018). For more complex movement analyses, tracking anatomical landmarks over time and space in video footage can provide data on kinematic inputs (e.g. position, stride) and outputs (e.g. speed, acceleration) during behaviours such as running or swimming. While this can be done manually (Puig‐Diví *et al*., 2019), freely available open‐source software can now automatically annotate, with review and correction then provided by researchers (Hedrick, 2008). The addition of machine learning techniques has improved accuracy and ease of feature tracking in both single (Mathis *et al*., 2018; Graving *et al*., 2019) and stereo video configurations (Klasen & Steinhage, 2022). Furthermore, drones offer the advantage of recording video from considerable distances, using a variety of angles and the ability to follow subjects in motion, making them a highly flexible tool for feature tracking in wildlife biomechanics.

### (2) Trends and implications

Similar to measurements of morphometrics, kinematic measurements *via* drones have been primarily accomplished for cetaceans (*N* = 9) and other marine species (*N* = 3). For baleen whales, drone‐derived morphometric measurements of different body parts, such as body length, gape angle and buccal cavity volume have been combined with aerial videos of foraging and swimming behaviour and prey distributions. This has yielded information on the biomechanics of different feeding activities and their consequent constraints on foraging capabilities (Werth *et al*., 2019; Torres *et al*., 2020). By further analysing drone video of lunge‐feeding events to extract fine‐scale kinematics of movement such as acceleration, head inclination, and body roll, as well as krill response distance and time to foraging whales, previously difficult‐to‐study predator–prey dynamics can be examined along with the energetic efficiencies of surface feeding events (Torres *et al*., 2020). Expanding further, if drone SCP morphometrics of multiple species are measured for baleen whales tagged with biologgers and animal‐borne cameras which directly measure kinematics during swimming (e.g. thrust power, Froude efficiency) and foraging (e.g. filtration time, engulfment capacity), the constraints of body size on the energetic efficiencies and fine‐scale kinematics of routine activities can be examined in detail for the largest extant animals on Earth (Gough *et al*., 2019, 2021, 2022; Kahane‐Rapport *et al*., 2020; Cade *et al*., 2023).

Anatomical feature‐tracking in drone‐borne video of swimming behaviours has also enabled the measurement of fine‐scale kinematics of movement, such as wingbeat frequency in manta rays (*Manta birostris*) (Fong, Hoffmann & Pate, 2022) and tailbeat frequency, body flexion, and swimming speed in blacktip sharks (*Carcharhinus limbatus*) (Porter, Ruddy & Kajiura, 2020). Such measurements of volitional movements from wild populations, which historically may have been derived from captive individuals (Lowe, 1996; Fish *et al*., 2016), help bridge gaps in understanding key aspects of movement ecology, such as predator–prey interactions and their physiological foundations (Nathan *et al*., 2008). Such measures can also be used for studies on comparative physiology, even amongst extinct taxa. For example, Farlow *et al*. (2018) utilised drone video to analyse the footfall pattern and biomechanical movement of an American crocodile (*Crocodylus acutus*) bottom‐walking on the sea floor. The analysis was then used to infer information about taxonomically similar extinct species from fossil trackways. This innovative approach allowed for comparative studies across species and geological epochs, bridging the realms of comparative physiology and paleobiology (Farlow *et al*., 2018).

While predominately used in marine environments, drones have also effectively measured kinematics in terrestrial species. Exemplified by Basu *et al*. (2019), a laterally positioned multirotor drone recording video alongside running giraffes (*Giraffa camelopardalis giraffa*) was used to estimate their speed, and through feature tracking, fine‐scale running kinematics including posture, gait, stride length and frequency, duty factor, and contralateral limb phase. The integration of SfM‐derived terrain features and manually validated morphometrics enhanced the precision of measurements and allowed calibration of kinematic measurements derived from drone video recordings (Basu *et al*., 2019).

### (3) Challenges and opportunities

Challenges in using drones for studying wildlife kinematics and biomechanics involve validating drone‐derived measurements with those from other methods like accelerometers, especially in species where kinematics are little studied or biologger attachment is difficult. Despite this, drone‐derived kinematic measurements of blue whale (*Balaenoptera musculus*) feeding behaviours align with measurements derived from tagging studies (Torres *et al*., 2020). Kinematic studies employing accelerometers are often validated using videos of animal behaviours (Ladds *et al*., 2018) and so aerially observable behaviours may be suited to drone validation. Such video validation requires aquatic species to be in relatively clear water with a contrast to background environments for distinguishing anatomical landmarks (Porter *et al*., 2020). By contrast, terrestrial species need to be either fully visible from above, or filmed laterally to reveal lower limb movements (Basu *et al*., 2019). A potential advancement in terrestrial environments may be the integration of LiDAR and millimetre wave radar sensors with machine learning, which has been successfully trialled for kinematic motion and pose tracking (joint detection and movement tracking) in humans, although not yet from drones (Sengupta *et al*., 2020; Li *et al*., 2022). Such systems would be robust to the effects of distance, lighting, and occlusion from vegetation on feature tracking, making them potentially ideal for kinematic analyses of wild terrestrial animals (Shield *et al*., 2023).

Further, recent advancements have included using drones as deployment systems for biologgers on cetaceans. While early attempts faced challenges, recent successes in deploying suction‐cup‐attached biologgers from drones to blue and fin whales (*B. physalus*) demonstrate extended data‐collection capabilities (Murakami *et al*., 2021; Wiley *et al*., 2023). This innovative approach overcomes traditional limitations, and reduces disturbance to target animals, marking a significant advance in biologger deployment.

## VI. BIOENERGETICS

### (1) Background

Within the field of wildlife ecology, bioenergetics provides a fundamental tool to quantifying how animals acquire, process, and utilise energy, providing information on an organism's ecological role, its position in food webs, and the resources needed for its growth, reproduction, and survival (Lavigne *et al*., 1982). This provides the foundation for comprehending how environmental stressors might impose additional energetic costs and how energy availability and quality can impact population dynamics (McHuron *et al*., 2022). The collection of bioenergetic data such as field metabolic rates and energy budgets has typically required invasive methods such as respirometry, the injection of radioactively labelled water, body condition *via* morphometrics, and animal‐borne cameras and/or biologgers (Williams *et al*., 2021; McHuron *et al*., 2022). In field settings, such methods may require physical or chemical immobilisation for safe application, followed by relocation and recapture of focal individuals to collect data at different time points or retrieve devices. These constraints generally limit research to small sample sizes and a subset of species (McHuron *et al*., 2022). While the use of drones for such research comes with its own limitations, recent studies demonstrate effective application for these purposes.

### (2) Trends and implications

Bioenergetic studies utilising drones have been largely limited to marine mammals, primarily cetaceans (*N* = 23), gray seals (*Halichoerus grypus*) (*N* = 1), and polar bears (*Ursus maritimus*) (*N* = 2) (Table S3). As examined in Section IV above, SCP has often been used to make repeated measurements of individual cetacean morphometrics over time, to calculate changes in body condition as a measure of growth or depletion of energetic reserves during critical life stages or various timeframes (e.g. Christiansen *et al*., 2016a, 2018, 2022b; Lemos *et al*., 2020a; Aoki *et al*., 2021a; Russell *et al*., 2023a). In gray seals, SfM‐derived 3D models have been used to quantify the changes in body volume of hundreds of mother–pup pairs concurrently, enabling the calculation of mass and energy transfer dynamics to be assessed for much larger sample sizes than possible when compared to traditional methods (Shero *et al*., 2021). Such scaling highlights a major utility of drones for ecophysiological studies, which are otherwise often limited in sample size by the invasive and time intensive nature of restraining and sampling individuals by hand.

The unique aerial perspective of drone‐captured video can also be used to investigate the energetic consequences of behaviours with little disturbance to subjects. Respiratory rates, nursing rates, and swimming speeds of cetaceans quantified from drone videos, combined with drone‐based morphometric measurements, and coupled with known energetic consequences for such activities, have delivered new information on the energetic expenditure of cetaceans during growth and development (e.g. Nielsen *et al*., 2019; Ejrnæs & Sprogis, 2021; Christiansen *et al*., 2023). This technology has also captured predation events (Azizeh *et al*., 2021) and anthropogenic encounters such as whale‐watching activities (Sprogis *et al*., 2023). In a terrestrial system, aerial videos of polar bear predation on seabird eggs have been used to estimate both the metabolic costs (e.g. foraging) and benefits (e.g. caloric gain) of consuming these resources, providing information on the ecophysiological consequences of a behaviour likely increasing due to climate change (Jagielski *et al*., 2021b). In a rare drone‐based study of avian ecophysiology, footage was used to assess the impact of human recreation on mute swans (*Cygnus olor*). The distance swans travelled when disturbed by kayakers was calculated, before pairing these data with metabolic rates to quantify the energetic costs of anthropogenic disturbance (Clausen *et al*., 2020). While such studies are pioneering, the expanding field of drone‐based animal behaviour studies promises similar insights for diverse species (Schad & Fischer, 2023).

### (3) Challenges and opportunities

Akin to the challenges faced in studying morphometrics or kinematics, employing drones for bioenergetic studies demands precise validation of measurements against established methods. This validation typically involves comparing drone‐derived measures of body condition, respiration rates (breaths per unit time), and energy expenditure (e.g. oxygen consumption) with expected values from the literature or directly measuring and comparing individuals within the study population. This process requires a thorough understanding of the target species, as well as adequate resources and expertise for implementing validation techniques (Leslie *et al*., 2020; Shero *et al*., 2021). Consequently, marine mammal researchers, who often face significant logistical challenges in applying direct methods for estimating energetic consumption (e.g. doubly labelled water), have been early adopters of drone‐based methods for bioenergetic assessments (Lifson & McClintock, 1966; McHuron *et al*., 2022). Additionally, the unique characteristics of cetaceans, such as the large and visible plumes of respiratory vapour they produce, make them particularly well‐suited for quantifying respiratory rate and inferring field metabolic rate using drone video (Folkow & Blix, 1992).

Creating activity budgets from aerial drone footage is feasible for various species (Schofield *et al*., 2022), but linking these activities to energetics requires detailed knowledge of their metabolic consequences. Studying the energetic costs or benefits of specific behaviours demands focused observations, understanding the caloric content of consumed food items, and knowledge of field metabolic rates, ideally for individuals of the same age, sex, and size class and measured in similar environmental conditions. As a tool to overcome such challenges, advances in drone hardware and software hold the potential for remote measurement of vital rates which can in turn be used to infer field metabolic rates for diverse species (Yazdi, Kilian & Culik, 1999; Green, 2011; Christiansen, Rasmussen & Lusseau, 2014). Drone video monitoring, employing high‐resolution zoom‐capable RGB (red‐green‐blue or standard colour) or thermal infrared (TIR or thermal) cameras, could capture breathing patterns in ungulates (Mufford *et al*., 2021) and marine reptiles (Reina *et al*., 2005; Robinson *et al*., 2020). Advances in fine‐scale feature extraction and video magnification might also enable the measurement of respiratory and heart rates through analysis of video alone (see Section VII).

## VII. VITAL SIGNS: RESPIRATION, HEART RATE, AND TEMPERATURE

### (1) Background

In the study of wildlife ecophysiology, monitoring vital signs like heart rate, respiratory rate and cycle duration, and animal temperature is typical, mirroring practices in human medical and veterinary fields to infer physiological conditions. Traditionally, these indicators are gathered through capture, handling, or biologgers, either implanted internally or worn externally (MacDonald *et al*., 2021). However, the advent of drones has enabled non‐contact measurement techniques for these metrics, particularly in cetaceans.

The use of drones for non‐contact research on vital sign detection has so far relied on detailed analysis of behaviours such as respiration in RGB video or equipping platforms with TIR sensors, which remotely gauge thermal radiation emitted by objects. TIR enables the remote measurement of surface temperatures without direct handling or invasive procedures, negating the need for traditional thermometers if surface temperatures can be reliably related to internal temperatures (McCafferty, 2007; Lathlean & Seuront, 2014; Mota‐Rojas *et al*., 2022). The integration of higher resolution thermal cameras into a growing array of commercial drone models highlights ongoing innovation and increasing accessibility of these tools for ecophysiological research.

### (2) Trends and implications

Beyond the quantification of respiration rates of cetaceans from counts of blow events during video focal follows, drones have also enabled the fine‐scale analysis of cetacean respiratory physiology (Martins *et al*., 2020). Combined SCP‐derived measures of blowhole relative area, in‐video quantification of respiration cycle duration (duration of a single breath) and seawater influx, along with drone‐collected blow samples (see Section VIII) have provided information on the respiratory dynamics of humpback (*Megaptera novaeangliae*) and North Atlantic right whales (*Eubalaena glacialis*) (Martins *et al*., 2020). TIR‐equipped drones have expanded this understanding by capturing heart and respiratory rates from aerial footage and measuring temperatures of dorsal fins and blowholes (Horton *et al*., 2019). Notably, high‐resolution time‐series analyses of blowhole thermal signatures have allowed researchers to infer apnoeic heart rates based on cyclical heat gain and loss patterns, which reflect cardiovascular function (Horton *et al*., 2019). Furthermore, a combination of drone‐TIR video and histological studies on tissue warming rates has been employed to examine heat loss patterns in North Atlantic right whales, allowing qualitative study of how thermal physiology affects observed heat anomalies in TIR (Lonati *et al*., 2022).

Drones have also aided in mapping the thermal environment to help determine its influence on physiology. High‐resolution drone‐derived models, such as SfM orthomosaics and digital elevation models, have captured fine‐scale surface temperature variations and their potential effects on intertidal species' survival within microhabitats (Choi *et al*., 2019) and the effects of environmental variation (e.g. distance to water and vegetation cover measured in orthomosaics) on thermoregulation strategies exhibited by viviparous lizards (*Zootoca vivipara*) (Rozen‐Rechels *et al*., 2021). In another approach, a multispectral sensor‐equipped drone was used to map vegetation cover using the normalised difference vegetation index (an index of vegetation greenness), along with logger‐based temperature data and in‐hand measurements, enabling assessment of the quality of thermal refuges and their impact on the body condition of desert lizards (*Mesalina bahaeldini*) (Stark *et al*., 2022). SfM thermal orthomosaics, combined with temperature logger validation, have also been used to investigate how habitat features influence heat stress in Nile crocodiles (*Crocodylus niloticus*) (Viljoen *et al*., 2023b) and how Atlantic salmon (*Salmo salar*) and brook trout (*Salvelinus fontinalis*) of different age and size classes avoid temperature stress in freshwater streams (Morgan & O'Sullivan, 2023).

### (3) Challenges and opportunities

Despite its many promising features, employing drone‐borne TIR for physiological studies poses significant challenges. The accuracy of TIR temperature derivation relies on multiple external (e.g. distance, angle, temperature, humidity) and internal (e.g. material, metabolism, physiological state) factors, and limitations and best practices require meticulous consideration (Lathlean & Seuront, 2014; Burke *et al*., 2019). TIR provides superficial temperatures, not always indicative of internal states, necessitating caution in correlations without species‐specific calibration studies (McCafferty, 2007). Thermal sensors are also relatively costly, with the highest resolution drone‐borne sensors currently priced above USD$20,000.

Software approaches using RGB drone sensors may provide cost‐effective alternatives to additional hardware such as TIR. For example, Eulerian video magnification (EVM) enables extraction of physiological information from standard video. EVM magnifies subtle colour or motion changes imperceptible to humans, enabling the isolation and amplification of skin colour fluctuations related to heart rate and body movement associated with breathing (Lauridsen *et al*., 2019). Although mathematically intricate, EVM can be applied to low‐resolution and unrestrained animal videos, offering a versatile approach for heart and respiratory rate determination (Lauridsen *et al*., 2019). Further, several recent studies have combined imaging photoplethysmography, the study of the interaction of light with biological tissues, with signal processing to obtain heart and respiratory rates of human subjects from drones (Al‐Naji, Perera & Chahl, 2017; Al‐Naji *et al*., 2019; Conte *et al*., 2021). While challenges related to camera and subject motion persist, such advancements showcase the potential of drones to aid in the study of wildlife health, disease dynamics, and physiological responses.

## VIII. MICROBIOME, ENDOCRINOLOGY, AND GENETICS

### (1) Background

Anthropogenic changes to the environment, including habitat fragmentation, climate change, pollution, and extractive exploitation of wildlife populations, have large implications for the health of wild animal and human populations (Acevedo‐Whitehouse & Duffus, 2009). Such pervasive pressures have been shown to disrupt endocrine and immune function, decrease reproductive capabilities, and lead to immunosuppression in wildlife (Cooke *et al*., 2021). Consequently, effective tools for the gathering of baseline information and on‐going monitoring of wildlife health have become a research and management priority (MacKenzie & Jeggo, 2019). Drones have been particularly useful for direct sample collection from cetaceans in the fields of microbiomes, endocrinology, and genetics.

Historically, obtaining health data from live, free‐ranging cetaceans has been challenging, but more recently, researchers have successfully sampled whale biological material remotely *via* blow (Hunt *et al*., 2013). The first published study using a drone for physiological data collection on wildlife emerged from this field in 2010, when a modified small remote‐controlled helicopter was used to carry several sample collection plates for microbiome sampling of several species of free‐ranging whales (Acevedo‐Whitehouse, Rocha‐Gosselin & Gendron, 2010). This method has now been applied to eight studies in nine cetacean species, most frequently to sample microbiomes as well as hormones and genetic material (Table S3). To obtain samples from blow, a drone is customised to carry attached sample plates (e.g. petri dishes, with or without remote opening/closing mechanisms), flown behind a surfacing cetacean from a nearby crewed vessel, and through the blow to collect a physical sample. As best practice, independent samples of surrounding air and sea water should be obtained to differentiate environmental or contaminant microbial communities from that of target organisms, a technique so far widely replicated (Table S3) (Pirotta *et al*., 2017; Atkinson *et al*., 2021).

### (2) Trends and implications

Whale blow samples collected by drones have enabled the identification of viral, bacterial, and archaeal communities to the species level, using techniques such as polymerase chain reaction (PCR), meta‐transcriptomics, and meta‐genomics (Table S3). This approach has been applied to various whale and dolphin species, allowing both inter‐ and intra‐species comparisons of microbiome communities, screening for potentially pathogenic bacteria, and the discovery of novel viral species (Acevedo‐Whitehouse *et al*., 2010; Apprill *et al*., 2017; Pirotta *et al*., 2017; Geoghegan *et al*., 2018; Centelleghe *et al*., 2020). Such data on microbiomes can provide a key biomarker for monitoring respiratory health in cetaceans, whose role as sentinel species makes them indicators of disturbances to ocean ecosystem functioning (Nelson *et al*., 2015).

Drones have been used in endocrine analyses of whales in eight studies, primarily through the analysis of blow samples collected *via* attached plates, which have detected hormones such as aldosterone, cortisol, progesterone, and testosterone in humpback and blue whales (Atkinson *et al*., 2021). While this direct sampling technique is limited to cetaceans, other techniques for drone‐assisted endocrine analyses pioneered for cetaceans may also be feasible for other taxa. SCP‐derived morphometrics such as length and width have been combined with external faecal sampling from the water to relate body condition indices to measured concentrations of faecal steroid hormones (progestins, androgens, glucocorticoids) (Lemos *et al*., 2022b; Pirotta *et al*., 2023) or pregnancy markers in known individuals (Pallin *et al*., 2022; Bierlich *et al*., 2022; Fernandez Ajó *et al*., 2023). Elsewhere, SCP used to measure body width at 50% of total body length was better at discriminating pregnant from non‐pregnant Pacific gray whales (*Eschrichtius robustus*) than metabolites found in faeces during the summer foraging period for this population (Fernandez Ajó *et al*., 2023). Drones may also be useful to obtain data on the physiological impact of anthropogenic stressors (e.g. elevated concentrations of glucocorticoid metabolites in faeces), whilst simultaneously accounting for factors that may confound hormone concentrations at the level of the individual (e.g. age, sex, or nutritional state). For example, individual faecal glucocorticoid metabolite samples were temporally matched with body condition indices and demographic units derived from drone imagery to account for individual variation when analysing the effect of vessel noise on gray whales (Lemos *et al*., 2022a).

In a pioneering study, drones have also been utilised for the direct sampling and extraction of genetic material from whale blow through simultaneous microbiome and endocrine sampling (Atkinson *et al*., 2021). This enabled the generation of nuclear and mitochondrial DNA (mtDNA) profiles, which were used for sex identification, haplotyping, and microsatellite genotyping of free‐ranging humpback whales, blue whales, and killer whales (*Orcinus orca*) (Atkinson *et al*., 2021). Although the number of successful mtDNA haplotype sequenced and sex‐matched samples was relatively low (on average 54%, and 39%, respectively), as well as the number of microsatellite loci amplified (on average 1.6 loci per sample), the ability to collect genetic information from non‐invasively obtained blow *via* drone marks a milestone in remote ecophysiological sampling.

### (3) Challenges and opportunities

While drone collection of microbiome, endocrine, and genetic material from wild cetaceans has yielded valuable data, several challenges with the application of this technique have been identified. Researchers have encountered difficulties obtaining sufficient material to support the necessary field or laboratory‐based analytical approaches, which are generally destructive in nature (Apprill *et al*., 2017; Atkinson *et al*., 2021). However, a recent study utilising a drone for genetic sampling of humpback and fin whales found that samples obtained from multiple blow events of the same whale, and those with larger and more abundant droplets, resulted in greater concentrations of mtDNA and more microsatellite loci amplified per sample (on average 7.5 loci), as well as a greater percentage of samples that could provide sexual identification and determine mtDNA haplotypes (89% and 80%, respectively), than previous studies (O'Mahony *et al*., 2024). Sampling from smaller cetacean species such as bottlenose dolphins (*Tursiops aduncus*) and Australian humpback dolphins (*Sousa sahulensis*), have posed additional challenges due to their limited blow production and less predictable behaviour, making drone sampling logistically challenging (Raudino *et al*., 2019; Robinson & Nuuttila, 2020). To address this, researchers have used drones with sample collection plates suspended by rope successfully to sample bottlenose dolphin blow (Centelleghe *et al*., 2020). However, matching samples to individual animals remained difficult as the species typically surfaces in groups (Centelleghe *et al*., 2020). Challenges for endocrine analyses include the need for biological and analytical validation of hormone assays, which are species and technique specific, including new methods like drone‐based sampling (Burgess *et al*., 2016). Despite these challenges and that they are currently limited to blow collection in cetaceans, non‐invasive sampling of microbiomes, endocrinology, and genetics is likely to receive ongoing attention.

Over the past several decades, there has been increasing use of drones to sample the environment directly (Ollero *et al*., 2021). Advances in the use of drones for industrial inspection purposes have recently been extended into the biological realm, giving rise to drones capable of sampling environmental DNA (eDNA) – genetic material present in environmental samples such as soil and water – from both terrestrial and freshwater environments (Doi *et al*., 2017; Aucone *et al*., 2023). Drones can also facilitate sample collection by enabling focal tracking of animals, allowing for fresh faecal samples to be obtained from cryptic species such as killer whales (Baird *et al*., 2022). Additionally, drones offer the potential for monitoring and detecting indicators of wildlife health, such as fine‐scale environmental conditions, pollutants, pathogens, and external parasites, in remote and inaccessible environments (Schilling *et al*., 2022).

Opportunities further emerge in the transport and on‐board analysis of biological samples using drones, a technique experiencing rapid uptake for human medical purposes, including microbiology and disease surveillance (Poljak & Šterbenc, 2020). Trials have shown that drones as sample transport mechanisms significantly reduce transport time without compromising biological parameters (Amukele *et al*., 2015; Sylverken *et al*., 2022). Drones have also been trialled as mobile laboratories. By temporarily replacing the propellers with snap‐on 3D‐printed centrifuge attachments, commercial multirotor drones have been successfully modified for on‐the‐ground sample preparation comparable to benchtop systems (Priye *et al*., 2016). Further, attachment of laboratory analysis devices to multirotors has allowed for on‐board capillary electrophoresis (Drevinskas *et al*., 2020), and in‐flight PCR analysis (Priye *et al*., 2016; Abid *et al*., 2021) to be conducted, with additional equipment costs as low as USD$50, utilising readily available components (Priye *et al*., 2016). As these techniques continue to be refined, they will likely overcome challenges in sample transport, storage, and analysis at remote field sites, bridging the gap between the field and the laboratory.

The potential for drones to collect biological samples directly from various species, extending beyond whale blow, is near. Robotic manipulators on multirotor drones have successfully collected samples from inaccessible and endangered plants in forest canopies and on otherwise inaccessible cliffs (Krisanski *et al*., 2022; La Vigne *et al*., 2022). This manipulator technology may also enable non‐invasive collection of biological samples (e.g. faeces, feathers, fur) from wildlife dwellings such as cliff or canopy nests or inaccessible carcasses. Drone‐assisted microbiome assessment from faecal samples of dangerous, elusive, or inaccessible species is a viable option, although validation is necessary due to potential degradation of sample quality caused by time, weather, and natural contamination (Menke, Meier & Sommer, 2015). Ultimately, the feasibility of direct physical sampling of live wildlife from drones *via* robotic manipulators hinges on improvements in safety, appropriate approach distances and minimising disturbance to wildlife. Ongoing explorations in soft robotics and biomimetic drone designs inspired by aerial animals may provide solutions to these challenges, potentially revolutionising physiological sampling for wildlife research (Tanaka *et al*., 2022).

## IX. DISEASE AND INJURY

### (1) Background

Emerging pathogens and infectious zoonotic disease are recognised as a major threat to both human health and biological diversity globally (Smith, Acevedo‐Whitehouse & Pedersen, 2009; Wille & Barr, 2022) and consequently the need for improved monitoring techniques has become a global research priority (MacKenzie & Jeggo, 2019). Recent attention has also been directed towards studying the impact of injury on wildlife, considering its implications for stress, growth, reproduction, and the potential for significant morbidity and mortality in certain populations (Perez‐Venegas *et al*., 2021; Knowlton *et al*., 2022; Reed *et al*., 2024). Ethical considerations for wildlife welfare in human‐managed landscapes further highlight the importance of monitoring and understanding the effect of injuries on wildlife (Pyke & Szabo, 2018). To meet these needs, researchers have used drones for monitoring disease and injury, employing remote sensing with RGB and/or TIR sensors to document health dynamics in diverse wildlife populations.

### (2) Trends and implications

The study of wildlife diseases using drones has been applied equally in terrestrial (*N* = 5) and marine (*N* = 5) environments, contrasting with the prevalence of applications in marine environments across most other applications of relevance to wildlife ecophysiology. In terrestrial environments, the ability of drones to operate at low altitudes to produce highly detailed spatial products, such as georeferenced SfM‐derived orthomosaics of landscapes, has been helpful for examining spatial epidemiology. For example, SfM‐generated orthomosaics showing forest cover over time were correlated with movement data from satellite telemetry of pig‐tailed macaques (*Macaca nemestrina*) and long‐tailed macaques (*M. fascicularis*) to investigate the impact of deforestation on the transmission of the malaria parasite *Plasmodium knowlesi*, both within macaque populations and to humans (Fornace *et al*., 2014; Stark *et al*., 2019).

Aerial photographs from drones have been used to inform spatially explicit maps detailing population counts of wild ungulates and domestic cattle along with habitat composition and the location and prevalence of disease cases (Barasona *et al*., 2014; Laguna *et al*., 2018). These studies highlighted how variations in habitat composition can influence overcrowding and the transmission of diseases like tuberculosis, underscoring the capability of drones to assess disease transmission risks at fine spatial and temporal scales. Another study examined the efficacy of using drone‐borne TIR for detecting and measuring the temperatures of wild boar (*Sus scrofa*) carcasses under various environmental conditions, to inform carcass removal actions that limit the spread of African swine fever (Rietz *et al*., 2023).

In the marine environment, aerial videos have been used to examine skin condition in fin whales for injury and potential disease assessment (Herr *et al*., 2023), and SCP has aided in the detection and quantification of dermatopathic diseases in humpback whales (Leslie *et al*., 2022). By repeatedly photographing afflicted individuals through time, the percentage surface area coverage of a skin disease can be calculated, allowing its progression to be monitored. Notably, there was a high level of agreement in disease diagnosis in these animals between assessments conducted from boats and drones, providing an effective validation for the use of drones for monitoring dermatopathic diseases (Leslie *et al*., 2022). The collection of blow‐samples from wild cetaceans (as described in Section VIII), has also allowed researchers genetically to screen respiratory microbiota for known pathogenic bacteria (Acevedo‐Whitehouse *et al*., 2010; Apprill *et al*., 2017; Pirotta *et al*., 2017).

Drones have also allowed insights into both the prevalence (*N* = 3) and effects of injuries (*N* = 6) on wildlife. SfM‐derived orthomosaics of seal colonies have been used to determine the prevalence of pinniped entanglement in marine debris (Claro *et al*., 2019; Martins *et al*., 2019) and drone‐based photographic monitoring of entanglement scars on cetaceans has enabled similar prevalence estimates (Ramp *et al*., 2021; Herr *et al*., 2023). A common conclusion of these studies was that drones improved entanglement prevalence estimates compared to ground or boat‐based surveillance, but that thin and transparent entanglements (e.g. monofilament fishing lines), were undercounted in drone‐borne imagery.

Drones as a tool to study the effects of injuries have been limited so far to cetaceans. These studies have generally used SCP for body condition, age and injury assessments along with behavioural data, either from aerial focal follows or satellite telemetry, to examine the energetic consequences of predation (Azizeh *et al*., 2021) and satellite tagging (Charlton *et al*., 2023), as well as age‐specific behaviours influencing vessel strike (Stepanuk *et al*., 2021). Further, drones have been used to observe the adverse long‐term effects of debris entanglement on North Atlantic right whale growth and development, combining morphometrics measurements from long‐term piloted aircraft sightings with those made from drones, to compare the body condition of entangled whales or of calves born to entangled mothers (Stewart *et al*., 2021b). Given that marine debris entanglement affects a substantial portion of aquatic mammal, bird, and sea turtle species (Gall & Thompson, 2015), but the sub‐lethal effects of entanglements on life history, fitness, and population viability are not well understood (Senko *et al*., 2020), the use of drones for increased monitoring of the prevalance and chronic effects of entanglement is an area needing further exploration.

### (3) Challenges and opportunities

Building on the successes in direct sampling of wildlife health by drones, visual diagnosis of diseases or injury in diverse wildlife species using RGB and TIR‐drone imagery presents exciting opportunities for the future of wildlife health monitoring. For disease surveillance, this may be especially applicable in species susceptible to disease transmission due to large and/or dense breeding sites, as is the case with many pinnipeds (Gardner *et al*., 2022). Where disease is detected by the presence of moribund or dead individuals, the spatial epidemiology of diseases could be explored further using high‐resolution, RGB or thermal drone imagery (Wolfe *et al*., 2005; Vora, 2008; Mills, Gage & Khan, 2010). Expanding upon this, the integration of TIR could be used for the detection of the symptoms of disease (e.g. elevated nasal temperatures due to fever) or active injuries such as entanglements (e.g. elevated temperatures at wound sites due to inflammation and infection) (Mota‐Rojas *et al*., 2022). If integrated with autonomous image classification and machine learning algorithms, such techniques could be adopted for large‐scale monitoring of managed environments (Keshavamurthy *et al*., 2022). Fine‐scale drone mapping and monitoring efforts could also complement satellite‐based remote sensing of landscapes to identify environmental predictors of disease (e.g. Barasona *et al*., 2014) or habitat selection of zoonotic hosts (e.g. Stark *et al*., 2019). Together, these approaches could aid existing remote sensing research in the prediction and mitigation of future outbreaks (Teitelbaum *et al*., 2024). Drone surveillance of wildlife populations has real potential to aid wildlife managers with rapid responses for disease outbreak prevention and containment, entanglements and other injuries necessitating intervention.

The adoption of higher resolution RGB and TIR sensors, similar to other methods mentioned in this review such as the use of biologgers or capturing of DNA from blow samples, generates large and complex data sets that users must be able to store, manage, and analyse properly. To achieve these goals, leveraging existing knowledge and new developments, particularly from fields such as data science (Grémillet, Chevallier & Guinet, 2022), environmental informatics (Frew & Dozier, 2012) and artificial intelligence (Osco *et al*., 2021; Krishnan *et al*., 2023), will be essential. As advanced sensors and data‐gathering payloads for drones become more accessible, the application of such techniques to capitalise effectively on the associated influx of data will also require collaboration and knowledge exchange across research fields, highlighting the value of multidisciplinary approaches to ecophysiological studies of wildlife.

## X. CONCLUSIONS

(1) Since 2010, there has been considerable growth in the number of reports using drones for studying wildlife ecophysiology, likely driven by cost reductions, increased accessibility and method validation. As new technologies, such as thermal imaging become more widely available, it is expected that non‐invasive ecophysiological research on wildlife will continue to advance. However, there are notable disparities in the field, with a geographic bias in studies taking place in and being conducted by researchers in the Global North. This emphasises the need for ongoing efforts to increase accessibility of remote wildlife ecophysiology monitoring to the global scientific community.
(2) The ‘air gap’ between a drone and study animal poses challenges to obtaining observations due to potential animal disturbance at closer ranges required to achieve suitable sensor resolution or sampling efficacy. Consequently, there is a focus on visually assessable measurements like body condition and morphometrics. However, modifying drone systems, or pairing them with external sampling techniques, facilitates multidisciplinary approaches and simultaneous sampling of multiple ecophysiological parameters, helping to bridge this gap.
(3) To date, studies have mainly focused on mammals and the marine environment, with an emphasis on cetaceans. Drones offer unique advantages for studying the aquatic environment due to the absence of visual obstacles compared to terrestrial landscapes, improved visibility of underwater attributes or behaviours from an aerial *versus* vessel‐ or land‐based perspective, and a lower degree of disturbance than approaching an animal in a vessel or in the water. Furthermore, investigator safety is increased by remotely monitoring from a vessel rather than entering the water. Finally, metrics obtained by directly capturing or sampling an animal, which is more tractable on land than in water, are more easily verified and require less calibration than remote measurements inferred from drones. For researchers studying marine wildlife, drones provide an effective solution for gathering data, especially for elusive or dangerous species, or where captivity is infeasible for ecophysiological research.
(4) Aerial photogrammetry (both SCP and SfM‐products) by drones facilitates morphometric measurements such as body length, mass, and volume, from large and difficult or impossible‐to‐capture animals. Integration of morphometrics with aerial videos and biologger data supports research into kinematics and biomechanics and bioenergetics of various terrestrial and marine species. Practitioners should review key lessons from marine mammal morphometrics and the larger field of photogrammetry science, which has developed best practices for image collection, calibration, measurement validation, and techniques to reduce and account for uncertainty in drone‐based measurements, as well as standard reporting protocols to enhance research quality and replicability.
(5) Direct sampling of whale blow *via* sampling plates attached to drones has proved effective for microbiome, genetic, and hormone analysis in various whale and dolphin species. Such samples have allowed the sequencing of microbiomes and viromes, hormone analysis, and screening of pathogens, and DNA extraction, contributing to health and disease monitoring and population management.
(6) The application of drone‐borne thermal imaging technology in physiological studies remains limited, but is increasing steadily as the technology becomes more accessible. Advancements have improved our understanding of cetacean thermal physiology, disease and injury detection, vital sign measurements, and enabled studies of thermal landscape quality and its effects on physiology. However, issues relating to the accuracy of perceived temperatures from different angles and altitudes and the effects of different environmental conditions persist.
(7) Advances in drone technology are rapidly transforming wildlife research in general, and in the field of ecophysiology, as demonstrated by this review. The integration of stereo‐video cameras, combining drone‐borne video with biologging data, and the use of drones as deployment systems for biologgers will extend capacity to measure wildlife form, movement and behaviour. Additionally, innovations in sample collection, transport, and on‐board processing *via* drones will enable a new frontier in wildlife health monitoring. Further advances in infrared thermal imaging, fine‐scale feature extraction and video magnification, and autonomous image classification have great potential for remote measurement of ecophysiological metrics and large‐scale monitoring of wildlife populations.
(8) Recommendations for the field include: enhancing research literacy and preparing for increased data influx and complexity from higher‐resolution sensors by fostering collaboration and leveraging advancements in remote sensing and data science; promoting interdisciplinary research to transcend traditional disciplinary boundaries by using new technologies and integrating drones with established physiological data‐collection methods; diversifying species and environment studies through the integration of methods and best practices from existing literature (Table S3); and using existing data repositories of drone‐borne imagery and videos to generate new information applicable to wildlife physiology research.
(9) The increasing sophistication and accessibility of drones and their sensors has established drones in the toolkits of both wildlife researchers and wildlife managers. Continuing improvements to the technology, methods of implementation and legislative frameworks will expand possibilities in the study of wildlife ecophysiology. Incorporation of the recommendations above will allow researchers to take advantage of both the anticipated and unexpected advancements to come, maximising research impact and furthering the field of wildlife ecophysiology.

## Supporting information


**Table S1.** Full list of search terms, filters, and search dates, and number of results following each search step from *Web of Science* and *Google Scholar* databases.
**Table S2.** Parameters collected from included studies during the data extraction stage of the scoping review.
**Fig. S1.** PRISMA flow chart detailing the process of record collection and study elimination for scoping review.
**Table S3.** Studies that used drones to collect ecophysiological data on wild animal species (organised taxonomically within class) in freshwater, terrestrial (coastal or inland), and marine environments.

**Data S1.** Full data set of extracted information for all papers included in this review.
