# Supplementary material for: Closing the air gap: the use of drones for studying wildlife ecophysiology
Source: Biol Rev Camb Philos Soc. 2025 Jan 17;100(3):1206–28. doi: 10.1111/brv.13181 (PMC12120397; doi:10.1111/brv.13181)
Supplement: Supplementary file 1 — Table S1. Full list of search terms, filters, and search dates, and number of results following each search step from Web of Science and Google Scholar databases. Table S2. Parameters collected from included studies during the data extraction stage of the scoping review. Fig. S1. PRISMA flow chart detailing the process of record collection and study elimination for scoping review. Table S3. Studies that used drones to collect ecophysiological data on wild animal species (organised taxonomically within class) in freshwater, terrestrial (coastal or inland), and marine environments. [file BRV-100-1206-s002.docx]

**Table S1.** Full list of search terms, filters and search dates, and number of results following each search step from *Web of Science* and *Google Scholar* databases. *Google Scholar* searches performed using Publish or Perish software with 1000 maximum result limit.

| Database | *Web of Science* | *Web of Science* | *Google Scholar* | *Google Scholar* (*via* Publish or Perish, 1000 search max) |
| --- | --- | --- | --- | --- |
| Search Date | 03 January 2024 | 22 July 2024 | 03 January 2024 | 22 July 2024 |
| Topic | drone OR drones OR UAS or RPAS or remotely piloted aircraft OR an unmanned aerial vehicle or unmanned aerial system | drone OR drones OR remotely piloted aircraft OR unmanned aerial vehicle OR unmanned aerial system OR unpiloted aircraft system OR unoccupied aerial system | drone OR drones OR UAS or RPAS or remotely piloted aircraft OR an unmanned aerial vehicle or unmanned aerial system | drone OR “remotely piloted aircraft” OR “unmanned aerial vehicle” OR “unpiloted aircraft system” OR “unoccupied aerial system” |
| All fields | physiology OR ecophysiology OR health OR stress OR injury OR disease OR body condition OR biomechanics OR kinematics OR energy OR thermoregulation | physiology OR ecophysiology OR health OR stress OR injury OR disease OR body condition OR morphometrics OR biomechanics OR kinematics OR energy OR thermoregulation OR metabolic rate OR bioenergetics OR growth OR respiration OR heart rate OR thermal | physiology OR ecophysiology OR health OR stress OR injury OR disease OR body condition OR biomechanics OR kinematics OR energy OR thermoregulation | physiology OR ecophysiology OR health OR stress OR injury OR disease OR body condition OR biomechanics OR kinematics OR energy OR thermoregulation |
| All fields | wildlife | wildlife OR animals OR vertebrate OR invertebrate OR terrestrial OR marine OR freshwater OR coastal | wildlife | wildlife |
| Number of results | 201 | 2255 | 980 | 980 |
| Not |  | *Apis mellifera* OR *Drosophila* OR zebrafish |  |  |
| Number of results with ‘Not’ filter |  | 1965 |  |  |
| Category filter |  | Environmental Sciences OR Ecology OR Genetics Heredity OR Toxicology OR Zoology OR Biology OR Physiology OR Marine Freshwater Biology OR Cell Biology OR Biodiversity Conservation OR Evolutionary Biology OR Neurosciences OR Behavioral Sciences OR Developmental Biology OR Entomology OR Fisheries OR Veterinary Sciences OR Oncology OR Environmental Studies OR Ornithology OR Virology OR Endocrinology Metabolism OR Cardiac Cardiovascular Systems OR Immunology OR Reproductive Biology OR Urology Nephrology OR Biophysics |  |  |
| Number of results with ‘Category’ filter |  | 634 |  |  |

**Table S2.** Parameters collected from included studies during the data extraction stage of the scoping review.

| **Category** | **Parameter collected** |
| --- | --- |
| **Bibliometric Data** | Authors, Title, Journal, Publication Year, Volume, Issue, Pages, DOI |
| **Research and lead author institution location(s)**  When assigning a country, all territories, islands, or dependencies were listed as part of their principal country. Geographic and political borders were used to define the country and continent, following the United Nations geoscheme (UNSD, 2022). These included Africa, Antarctica, Asia, Central America and the Caribbean, Europe, North America, Oceania, and South America. | The country and continent where the research was conducted and country and continent of the lead author’s institution.  If the research used data collected in multiple locations, or there was more than one lead author, all countries and continents were recorded. |
| **Topic(s)** | The relevant physiological topic or topics that were explored in each study as defined by six *a priori* research topic categories:  (1) *Body Condition and Morphometrics:* measurement of outward physical characteristics (e.g. size, girth, mass) to derive anatomical measurements or indices of body condition.  (2) *Kinematics and Biomechanics*: measurement of an animal’s physical motion or the structure, function, or mechanical aspects of its movement. Excludes tracking an animal’s movement through space or its interactions with other individuals (i.e. focuses on how it is moving, not what it is doing).  (3) *Bioenergetics*: measurement of acquisition and allocation of energy by individuals to support maintenance, activity, growth and reproduction.  (4) *Vital Signs: Respiration, Heart Rate, and Temperature*: measurement of respiration, heart rates and body or surface temperature, including physiological aspects of each.  (5) *Microbiome, Endocrinology, and Genetics*: sampling and analysis of biological materials to determine attributes of animal microbiome and/or virome, endocrine function, and/or genetics.  (6) *Disease and Injury*: determination of the effect or prevalence of disease and/or injury within a study animal or population. |
| **Environment(s)** | Environment the drone was used to collect data in:  (1) *marine* (all marine waters, including estuarine systems)  (2) *freshwater* (all freshwater bodies)  (3) *coastal* (land areas within 500 m of the high tide line)  (4) *inland* (terrestrial areas more distant than 500 m from any tideline. |
| **Species studied (class, functional grouping, species)** | The species studied. |
| **Type of drone** | The general category of drone system used from the following categories:  (1) Commercial Multirotor (off-the-shelf commercially available multi-rotor drone, not specified as modified).  (2) Modified or Custom Multirotor (commercially available drone modified for research purposes, modifications may include bespoke sensor mounts or external sample collection plates. Alternatively, a custom-built or ordered drone not available *via* direct retail).  (3) Commercial Fixed-Wing (off-the-shelf commercially available fixed-wing drone, not specified as modified).  (4) Modified or Custom Fixed-Wing (commercial fixed-wing drone modified for research purposes, using same criteria as above).  (5) Both or all types of Multirotor or Fixed-Wing (the study utilised some combination of the above categories). |
| **Drone make, model, and weight class***  *Drones were assigned to weight classes using definitions from the Australian Government Civil Aviation Safety Authority (CASA, 2022). CASA classifications were adopted as they provide a comprehensive weight categorisation system that can be translated into regulatory requirements for other nations. | The make and model of drone platforms used in the study and their unladen (i.e. body and battery weight only, excluding detachable payloads) and following weight classification*:  (1) Micro (< 250 g)  (2) Very small (250 g – 2 kg)  (3) Small (2 ­– 25 kg)  (4) Medium (25 – 150 kg)  (5) Large (< 150 kg) |
| **Sensors used** | The type category of sensors utilised in each study, e.g. standard colour Red-Green-Blue (RGB), Infrared Thermography (TIR), Light Detection and Ranging (LiDAR).  If the drone was not explicitly stated to have a sensor, it was assumed that a RGB visual sensor was available for navigational purposes. |
| **Additional drone-borne hardware** | Modifications made to the drone with any type of external hardware that was not specifically a sensor (e.g. sample collection plates for whale blow or vapour). |
| **Non-drone hardware used** | Additional hardware used to collect ecophysiological data that was not drone-borne. |
| **Technique and product created** | Any implemented analyses or product creation technique that relied on data collected from a drone. |
| **Metrics reported and source** | The reported metrics measured by the drone and/or non-drone technique used alone or in part to calculate or infer an ecophysiologically relevant endpoint. |
| **Ecophysiologically relevant endpoint calculated or inferred** | Any ecophysiologically relevant endpoint (e.g. body condition, demographic unit, field metabolic rate, foraging efficiency) calculated in the study. |
| **Calibration or correction method applied (if applicable)** | Whether a calibration or correction method was reported to have been used and if so, what it was. |


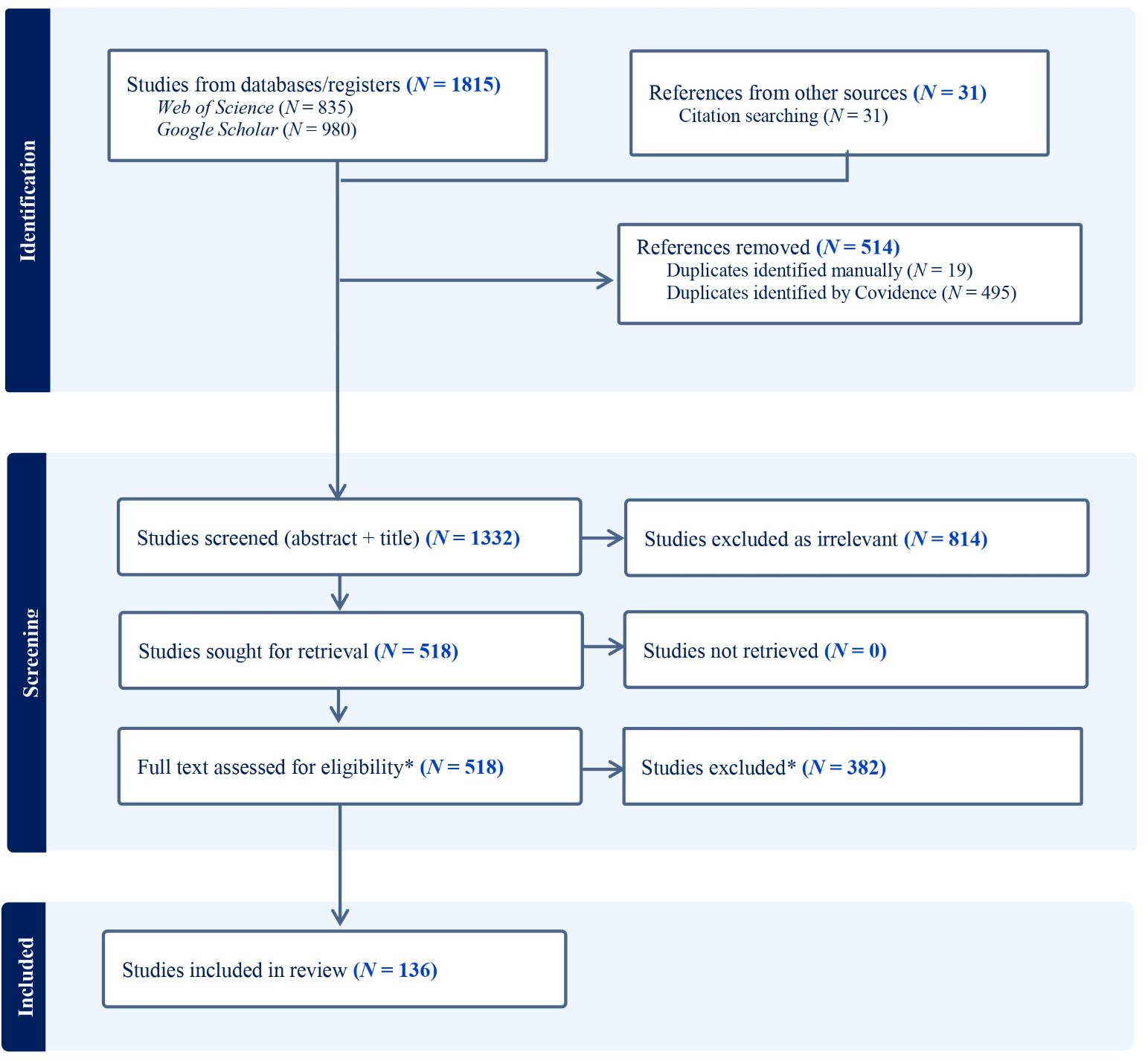


**Fig. S1.** PRISMA flow chart detailing the process of record collection and study elimination for scoping review. *See Table 1 for inclusion and exclusion criteria.

**Table S3.** Studies that used drones to collect ecophysiological data on wild animal species (organised taxonomically within class) in freshwater, terrestrial (coastal or inland), and marine environments. Type of drone used in the study (commercial multirotor; modified or custom multirotor; commercial fixed-wing; modified or custom fixed-wing; or a combination thereof) is specified, where ‘&’ indicates that a combination is used in the cited paper, no conjunction indicates the same type of drone was used in all cited studies, and superscripts (*,†) indicate when different types were used in each study. The drone or additional, non-drone-based technique or techniques (non-drone techniques indicated in *italics*) used to derive ecophysiologically relevant metrics are specified for each study with lower-case letter superscripts (e.g. a,b,c). The ecophysiological metrics measured by the drone in each study are indicated by numerical superscripts (e.g. 1,2,3), and the relevant ecophysiological calculation or inference made in the study are indicated by upper-case letter superscripts (e.g. A,B,C). ‘Total length’ refers to a total measurement of the body, whereas ‘body length(s)’ refers to a length measurement for a sub-unit of the body. A caret (^^^) superscript indicates the study did not report an applicable calibration or validation metric and a caron (ˇ) superscript indicates no calibration or validation metric was applicable to the technique used. No caret or caron indicates that a calibration or validation metric was reported for that study. For all columns, a lack of superscripts following a specific technique, metric, or relevant calculation or inference indicates this feature was shared between all studies on that species for that category. Abbreviations: DSM, Digital Surface Model; SCP, Single Camera Photogrammetry; SfM, Structure from Motion.

| **Species** | **Drone type(s) used** | **Technique and/or equipment used** | **Ecophysiological metric measured by drone** | **Relevant calculation or inference** | **References** |
| --- | --- | --- | --- | --- | --- |
| **Body Condition and Morphometrics** | | | | | |
| *Class: Scyphozoa, Marine* | | | | | |
| *Catostylus mosaicus*  Jelly blubber | Commercial multirotor | Photogrammetry (SfM – Orthomosaic) | Body widths, Surface area | Morphometrics | Raoult & Gaston (2018)^^^ |
| *Class: Chondrichthyes, Marine* | | | | | |
| *Bathytoshia brevicaudata*  Short-tail stingray | Commercial multirotor | Photogrammetry (SCP), Video | Body length, Body widths, Behaviour, Speed of movement | Morphometrics, Foraging dynamics | Oleksyn *et al.* (2020) |
| *Mobula alfredi*  Reef manta ray | Commercial multirotor | Photogrammetry (SCP) | Body length, Body widths | Morphometrics, Demographic units, Developmental ecology | Setyawan *et al.* (2022*a*); Setyawan *et al.* (2022*b*) |
| *Carcharodon Carcharias*  White shark | Commercial multirotor | Photogrammetry (SCP), Video | Total length, Speed of movement, Behaviour, Location^1^ | Morphometrics, Foraging dynamics | Tucker *et al.* (2021)^1^; Colefax *et al.* (2020) |
| *Ginglymostoma cirratum*  Nurse shark | Modified or custom multirotor | Photogrammetry (Stereo-video cameras) | Body lengths, Body widths | Morphometrics | Piacenza *et al.* (2022) |
| *Rhincodon typus*  Whale shark | Commercial multirotor | Photogrammetry (SCP), Video | Body length, Body widths, Dorsal surface area | Body condition | Whitehead *et al.* (2022) |
| *Class: Osteichthyes, Marine* | | | | | |
| *Thunnus thynnus*  Atlantic bluefin tuna | Modified or custom multirotor | Photogrammetry (SCP) | Total length, Body widths, Behaviour | Morphometrics | Jech *et al.* (2020) |
| *Class: Reptilia* | | | | | |
| *Freshwater* | | | | | |
| *Crocodylus acutus*  American crocodile | Commercial multirotor | Photogrammetry (SfM – Orthomosaic) | Total length | Morphometrics, Demographic units | Marín-Enríquez *et al*. (2023)^^^ |
| *Crocodylus palustris*  Mugger crocodile | Commercial fixed-wing | Photogrammetry (SfM – Orthomosaic), *Post-processing kinematics* | Total length | Demographic units | Sawan *et al.* (2023)^^^ |
| *Crocodylus niloticus*  Nile crocodile | Commercial multirotor | Photogrammetry (SfM – Orthomosaic) | Total length, Body lengths^1^, Body widths^1^, Surface area^1^, Body perimeter^1^ | Morphometrics^A^, Body condition^B^ | Ezat *et al*. (2018)^A ,^^; Viljoen *et al.* (2023*a*)^1,B,^^ |
| *Terrestrial: Inland* | | | | | |
| *Mesalina bahaeldini* | Commerical fixed-wing | Multispectral, Photogrammetry (SfM – Orthomosaic, Multispectral Orthomosaic, DSM), *Temperature loggers* | Habitat composition | Body condition, Thermal habitat quality | Stark *et al.* (2022) |
| *Marine* |  |  |  |  |  |
| *Chelonia mydas*  Green sea turtle | Commercial multirotor^*^, modified or custom multirotor^†^ | Photogrammetry (SCP^a^ & Stereo-Video Cameras^b^), *Satellite telemetry*^a^ | Body lengths, Body widths | Morphometrics, Comparative physiology^A^, Demographic units^A^ | Stokes *et al.* (2023)^*,a,A,^ˇ; Piacenza *et al*. (2022)^b,†^ |
| *Eretmochelys imbricate*  Hawksbill sea turtle | Commercial multirotor | Photogrammetry (SCP), *Satellite telemetry* | Body lengths, Body widths | Morphometrics, Comparative physiology, Demographic units | Stokes *et al.* (2023)ˇ |
| *Class: Mammalia* | | | | | |
| *Freshwater* | | | | | |
| *Hippopotamus amphibious*  Common hippopotamus | Commercial multirotor | Photogrammetry (SCP), *Multiple imputation*^a^ | Total length, Body lengths^1^, Body widths^1^, Behaviour | Demographic units, Body condition^A^ | Inman & Leggett (2022)^a,1,A^; Inman *et al.* (2019)^^^ |
| *Terrestrial: Inland* | | | | | |
| *Oryx leucoryx*  Arabian oryx | Commercial multirotor & commercial fixed-wing | Photogrammetry (SfM – Orthomosaic), AI/machine learning | Total length, Dorsal surface area, Body width | Morphometrics, Demographic units, Reproductive metrics | de Kock *et al.* (2021) |
| *Cervus elaphus*  Red deer | Commercial multirotor | Photogrammetry (SCP), Thermal | Total length, Body widths | Morphometrics | Larsen *et al.* (2023) |
| *Lepus sp.*  Hare sp. | Commercial multirotor | Photogrammetry (SCP), Thermal | Total length, Body widths | Morphometrics | Larsen *et al.* (2023) |
| *Martes foina*  Stone martin | Commercial multirotor | Photogrammetry (SCP), Thermal | Total length, Body widths | Morphometrics | Larsen *et al.* (2023) |
| *Meles meles*  European badger | Commercial multirotor | Photogrammetry (SCP), Thermal | Total length, Body widths | Morphometrics | Larsen *et al.* (2023) |
| *Bos taurus*  Domestic cow | Commercial multirotor | Photogrammetry (SCP), Thermal | Total length, Body widths | Morphometrics | Larsen *et al.* (2023) |
| *Elephas maximus*  Asian elephant | Commercial multirotor | Photogrammetry (SfM – Orthomosaic), Laser altimeter, Thermal, *Satellite telemetry, AI/machine learning* | Total length, Body widths, Abundance, Distribution | Body condition, Development, Demographics | Rahman *et al.* (2023) |
| *Terrestrial: Coastal* | | | | | |
| *Arctocephalus pusillus doriferus*  Australian fur seal | Commercial multirotor | Photogrammetry (SfM – Orthomosaic) | Total length, Body width | Body condition | Allan *et al.* (2019) |
| *Neophoca cinerea*  Australian sea lion | Commercial multirotor | Photogrammetry (SfM – Orthomosaic) | Total length, Body widths, Surface area, Body perimeter | Body condition | Hodgson *et al.* (2020) |
| *Halichoerus grypus*  Gray seal | Commercial multirotor^*^, modified or custom multirotor^†^ | Photogrammetry (SCP^a^ & SfM – Orthomosaic^b^, 3D model^b^, DSM^b^) | Total length, Behaviour, Body volume^1^ | Morphometrics^A^, Body condition^B^, Development^B^, Energy dynamics^B^ | Shero *et al.* (2021)^*,b,1,B^; Pomeroy *et al*. (2015)^†,a,A^ |
| *Phoca vituline*  Harbour seal | Modified or custom multirotor | Photogrammetry (SCP^a^ & SfM – Orthomosaic^b^), AI/Machine learning^c^ | Total length, Behaviour^1^, Body widths^2^, Surface area^2^, Body perimeter^2^ | Morphometrics, Demographics^A^ | Infantes *et al.* (2022)^b,c,2,A^; Pomeroy *et al.* (2015)^a,1^ |
| *Hydrurga leptonyx*  Leopard seal | Modified or custom multirotor | Photogrammetry (SCP) | Total length, Body widths, Body lengths^1^ | Morphometrics, Body condition^A^ | Krause *et al.* (2017)^1,A^; Goebel *et al.* (2015) |
| *Mirounga angustirostris*  Northern elephant seal | Commercial multirotor | Photogrammetry (SCP) | Body area | Morphometrics | Alvarado *et al.* (2020) |
| *Mirounga leonine*  Southern elephant seal | Commercial multirotor | Photogrammetry (SfM - Orthomosaic) | Body length, Surface area | Morphometrics, Demographics | Fudala & Bialik (2020) |
| *Marine* | | | | | |
| *Eubalaena glacialis*  North Atlantic right whale | Modified or custom multirotor | Photogrammetry (SCP), Laser altimeter | Total length, Body widths^1^, Body volume^2^, Injury assessment^3^ | Body condition^A^, Demographic units^B^, Comparative physiology^B^, Development^C^, Reproductive metrics^D^, Impact of external factor (injury)^E^ | Stewart *et al.* (2022)^C,D^; Stewart *et al.* (2021*b*)^1,3,A,C,E^; Christiansen *et al.* (2020*a*)^1,2,A,B^ |
| *Eubalaena australis*  Southern right whale | Commercial multirotor^*^, modified or custom multirotor^†^ | Photogrammetry (SCP), Video^a^, Laser altimeter^b^, *Manned aircraft*^c^, *Hydrophone*^d^, *Video cameras*^e^ | Total length, Body widths^1^, Body lengths^2^, Body volume^3^, Respiration rate^4^, Behaviour^5^, Speed of movement^6^, Injury assessment^7^, Nursing rate^8^, Skin condition^9^ | Morphometrics^A^, Body condition^B^, Demographic units^C^, Reproductive metrics^D^, Development^E^, Energy dynamics^F^, Impacts of external factor (disturbance^G^ & injury^H^), Comparative physiology^I^ | Charlton *et al*. (2023)^*,1,3,7,9.B,D,H^; Christiansen *et al.* (2023)^*,a,b,1,3.4,5,6,A,B,C,D,F^; Sprogis *et al*. (2023)^*,a,d,4,5,6,8,E,F,G^; Vermeulen *et al.* (2023)^*,1,c,B^; Christiansen *et al.* (2022*a*)^*,b,1,3,,B,C,E^; Christiansen *et al*. (2022*b*)^*,b,1,3,A,B,D,E^; Johnston *et al*. (2022)^†,b,1,2,A,B,C^; Christiansen *et al.* (2020*a*)^*,†,b,1,3,B,C,I^; Christiansen *et al*. (2019)^*,b,1,2,3,A^; Nielsen *et al*. (2019)^*,b,1,4,5,6,B,D,E,F^; Christiansen *et al.* (2018)^†,b,1,2,3,B,D,E^; Dawson *et al.* (2017)^†,b,1,A^ |
| *Eschrichtius robustus*  Gray whale | Commercial multirotor^*^, modified or custom multirotor^†^ | Photogrammetry (SCP), Video^a^, Laser altimeter^b^, Temperature compensated barometer^c^, *Manned aircraft*^d^, *Faecal sampling (Endocrine)*^e^ | Total length, Body widths^1^, Body lengths^2^, Surface area^3^, Body volume^4^, Behaviour^5^, Injury assessment^6^ | Body condition^A^, Demographic units^B^, Reproductive metrics^C^, Development^D^, Energy dynamics^E^, Impacts of external factor (disturbance ^F^, injury ^G^ ), Stress^H^, Comparative physiology^I^, Demographics^I^ | Bierlich *et al.* (2023*a*)^*,1,b,B,D,I^; Fernandez Ajó *et al.* (2023)^*,e,1,2,A,B,C^; Pirotta *et al.* (2023)^*,b,e,1,2,3,A,B,F,H^; Stewart *et al.* (2023)^†,1,b,c,d,A,J^; Lemos *et al.* (2022*b*)^*,e,1,2,3,A,E,H^; Lemos *et al.* (2022*a*)^*,a,b,e,1,2,5,6,A,F,H^; Torres *et al.* (2022)^*,a,b,d,2,4,6,A,I^; Christiansen *et al*. (2021)^*,a,b,1,2,4,5,A,B^; Lemos *et al.* (2020*a*)^*,a,1,2,3,5,A,B^; Lemos *et al.* (2020*b*)^*,a,e,1,2,,5,6,A,B,C,G,H^; Burnett *et al.* (2019)^*,1,3,4,A^ |
| *Balaenoptera bonaerensis*  Antarctic minke whale | Commercial multirotor^*^, modified or custom multirotor^†^ | Photogrammetry (SCP), Video^a^, Laser altimeter^b^, *AI/Machine learning*^c^, *Biologger*^d^, *Animal-borne cameras*^e^, *Tissue sampling (Genetic & Endocrine)*^f^, *Sonar*^g^ | Total length, Body widths^1^, Body lengths^2^, Surface area^3^, Behaviour^4^, Engulfment capacity^5^, Body volume^6^ | Morphometrics^A^, Body condition^B^, Demographic units^C^, Reproductive metrics^D^, Demographics^E^, Foraging dynamics^F^, Comparative physiology^G^, Kinematic scaling^H^, Genetic profile^I^, Fine-scale kinematics^J^, Energy dynamics^K^ | Cade *et al.* (2023)^*,a,b,d,e,2,4,A,F,G,H,J^; Gough *et al.* (2022)^*,†,a,b,d,e,1,4,5,A,F,G,H,J,K^; Pallin *et al.* (2022)^†,a,b,f,1,C,D,E,I^; Bierlich *et al.* (2021*a*)^*,†,b,1,3,6,A,B^; Gough *et al.* (2021)^*,†,b,d,e,1,2,3,A,G,H,J,K^; Savoca *et al.* (2021)^*,†,b,d,e,g,1,5,A,F,G,K^; Kahane-Rapport *et al.* (2020)^*,†,a,b,d,e,1,3,5,F,G,H^; Gough *et al.* (2019)^*,†,b,d,e,1,A,G,H,J^; Gray *et al.* (2019)^*,†,c,A,C^ |
| *Balaena mysticetus*  Bowhead whale | Commercial multirotor | Photogrammetry (SCP), Video^a^, Laser altimeter^b^ | Total length^1^, Body widths^2^, Body lengths^3^, Body volume^4^, Behaviour^5^, Skin condition^6^ | Body condition^A^, Demographic units^B^, Reproductive metrics^C^, Development^D^, Energy dynamics^E^, Foraging dynamics^F^ | Christansen *et al.* (2024)^b,2,3,4,A,B,C,D,E,F^; Fortune *et al.* (2017)^a,1,5,6,B,D^ |
| *Balaenoptera musculus*  Blue whale | Commercial multirotor^*^, modified or custom multirotor^†^ | Photogrammetry (SCP), Video^a^, Laser altimeter^b^, *AI/machine learning*^c^, *Biologger*^d^, *Animal-borne cameras*^e^, *Sonar*^f^ | Total length, Body widths^1^, Body lengths^2^, Surface area^3^, Behaviour^4^, Engulfment capacity^5^, Speed of movement^6^, Body volume^7^ | Morphometrics^A^, Body condition^B^, Demographic units^C^, Foraging dynamics^D^, Comparative physiology^E^, Kinematic scaling^F^, Fine-scale kinematics^G^, Energy dynamics^H^ | Barlow *et al.* (2023)^*,b,1,2,B,E^; Gough *et al*. (2022)^*,†,a,b,d,e,1,4,5,A,D,E,F,G,H^; Bierlich *et al.* (2021*a*)^*,†,b,1,3,7,A,B^; Gough *et al*. (2021)^*,†,b,d,e,1,2,3,A,E,F,G,H^; Savoca *et al*. (2021)^*,†,b,d,e,f,1,5,A,D,E,H^; Kahane-Rapport *et al*. (2020)^*,†,a,b,d,e,1,3,5,D,E,F^; Leslie *et al.* (2020)^†,2,A^; Torres *et al.* (2020)^*,a,g,1,4,6,D,G^; Burnett *et al.* (2019)^*,1,3,7,A,B^; Gough *et al*. (2019)^*,†,b,d,e,1,A,E,F,G;^ Gray *et al*. (2019)^*,†,c,A,C^, Durban *et al.* (2015*b*)^†,A^ |
| *Balaenoptera edeni brydei*  Bryde’s whale | Commercial multirotor^*^, modified or custom multirotor^†^ | Photogrammetry (SCP), Laser altimeter^a^, *Video cameras*^b^, *Biologger*^c^, *Animal-borne cameras*^d^ | Total length, Body widths^1^, Body lengths^2^, Surface area^3^ | Morphometrics^A^, Comparative physiology^B^, Kinematic scaling^C^, Energy dynamics^D^, Fine-scale kinematics^D^ | Gough *et al*. (2021)^*,†,a,b,c,d,1,2,3,A,B,C,E^; Gough *et al*. (2019)^*,†,a,b,c,d,1,A,B,C,D,E^ |
| *Balaenoptera physalus*  Fin whale | Commercial multirotor & modified or custom multirotor | Photogrammetry (SCP), Video^a^, Laser altimeter^b^, *Biologger*^c^, *Animal-borne cameras*^d^, *Sonar*^e^ | Total length, Body widths^1^, Body lengths^2^, Surface area^3^, Behaviour^4^, Engulfment capacity^5^ | Morphometrics^A^, Foraging dynamics^B^, Comparative physiology^C^, Kinematic scaling^D^, Fine-scale kinematics^E^, Energy dynamics^F^ | Gough *et al*. (2022)^a,c,d,1,4,5,A,B,C,D,E^; Gough *et al*. (2021)^b,c,d,1,2,3,A,C,D,E,F^; Savoca *et al*. (2021)^b,c,d,e,1,5,A,B,C,F^; Kahane-Rapport *et al*. (2020)^a,b,c,d,1,3,5,B,C,D^; Gough *et al*. (2019)^b,c,d,1,A,C,D,E^ |
| *Balaenoptera borealis*  Sei whale | Commercial multirotor & modified or custom multirotor | Photogrammetry (SCP), Laser altimeter, *Biologger*, *Animal-borne cameras* | Total length, Body widths, Body lengths, Surface area | Morphometrics, Comparative physiology, Kinematic scaling, Fine-scale kinematics, Energy dynamics | Gough *et al.* (2021) |
| *Megaptera novaeangliae*  Humpback whale | Commercial multirotor^*^, modified or custom multirotor^†^ | Photogrammetry (SCP^a^ & SfM – 3D model^b^), Video^c^, Laser altimeter^d^, *AI/machine learning*^e^, *Biologger*^f^, *Animal-borne cameras*^g^, *Sonar*^h^, *Tissue sampling (Genetic* ^i^, *Endocrine* ^i^, *Lipid concentration^j^)*, *Video cameras*^k^, *Handheld LIDAR*^l^ | Total length^1^, Body widths^2^, Body lengths^3^, , Body volume^4^, Surface area^5^, Behaviour^6^, Skin condition^7^, Respiration rate^8^, Speed of movement^9^, Engulfment capacity^10^, Location of anatomical landmarks^11^ | Morphometrics^A^, Body condition^B^, Demographic units^C^, Reproductive metrics^D^, Development^E^, Foraging dynamics^F^, Comparative physiology^G^, Kinematic scaling^H^, Energy dynamics^I^, Migratory costs^J,^ Disease surveillance^K^, Fine-scale kinematics^L^ | Russell *et al.* (2023*a*)^*,a,d,1,2,3,4,B,C,J^; Russell *et al.* (2023*b*)^*,a,d,1,2,3,A,B,G^; Bierlich *et al.* (2022)^*,†,a,d,i,1,2,3,4,B,C^; Chenoweth *et al.* (2022)^*,b,l,1,2,3,4,A,^ˇ; Russell *et al*. (2022)^*,a,1,2,3,4,B,C,E,J^; Ejrnæs & Sprogis (2021)^*,a,c,1,2,6,8,E,I^; Gough *et al*. (2022)^*,†,a,c,d,f,g,1,2,6,10,A,F,G,H,I,L^; Hirtle *et al.* (2022)^*,a,d,1,2,3,4,B,C^; Leslie *et al.* (2022)^*,†,a,5,7,K^; Savoca *et al*. (2021)^*,†,a,d,f,g,h,1,2,10,A,F,G,I^; Aoki *et al.* (2021*a*)^*,a,f,1,2,3,5,B,F,I^; Bierlich *et al*. (2021*a*)^*,†,a,d,1,2,5,A,B^; Gough *et al.* (2021)^*,†,a,d,f,g,1,2,3,5,A,G,H,I,L^; Ratsimbazafindranahaka *et al.* (2021)^*,a,1,2,A,E^; Christiansen *et al*. (2020*b*)^*,a,d,j,1,2,4,B,C,E,I^; Kahane-Rapport *et al.* (2020)^*,†,a,c,d,f,g,1,2,5,10,F,G,H^Gray *et al.* (2019)^*,†,a,e,1,A,C^; Gough *et al.* (2019)^*,†,a,d,f,g,1,2,A,G,H,L^; Werth *et al.* (2019)^*,a,c,k,1,2,3,6,9,11,A,F,L^^; Christiansen *et al.* (2016*a*)^*,a,1,2,5,B,C,D,E^ |
| *Physeter macrocephalus*  Sperm whale | Commercial multirotor | Photogrammetry (SCP), Laser altimeter, *Hydrophone*^a^ | Total length, Body widths^1^, Body lengths^2^, Body height^3^, Body volume^4^ | Morphometrics^A^, Body condition^B^, Demographic units^C^ | Glarou *et al.* (2023)^1,2,3,4,A,B,C^; Dickson *et al*. (2021)^a,A^ |
| *Pontoporia blainvillei*  Francsicana dolphin | Commercial multirotor | Photogrammetry (SCP) | Total length, Body widths | Morphometrics, Body condition | de Oliveira *et al.* (2023) |
| *Sousa sahulensis*  Australian humpback dolphin | Commercial multirotor | Photogrammetry (SCP) | Total length, Body widths | Morphometrics | Christie *et al*. (2021) |
| *Orcaella heinsohni*  Australian snubfin dolphin | Commercial multirotor | Photogrammetry (SCP) | Total length, Body widths | Morphometrics | Christie *et al.* (2021) |
| *Tursiops truncates*  Bottlenose dolphin | Commercial multirotor^*^, modified or custom multirotor^†^ | Photogrammetry (SCP), Laser altimeter, *Handheld laser photogrammetry*^a^ | Total length, Body widths^1^, Body lengths^2^ | Morphometrics^A^, Body condition^B^, Demographic units^C^, Demographics^D^, Reproductive metrics^E^ | Vivier *et al.* (2023)^*,†,2,A,C,D^; Cheney *et al.* (2022)^†,a,1,B,C,E^ |
| *Sotalia guianensis*  Guiana dolphin | Commercial multirotor | Photogrammetry (SCP) | Total length, Body widths | Morphometrics, Body condition | de Oliveira *et al.* (2023) |
| *Feresa attenuata*  Pygmy killer whale | Commercial multirotor | Photogrammetry (SCP), Laser altimeter, Video, *Tissue sampling (lipid concentration)* | Total length, Body widths, Body Volume, Behaviour | Body condition, Energy dynamics, Impacts of external factor (disturbance) | Currie *et al.* (2021) |
| *Orcinus orca*  Killer whale | Modified or custom multirotor | Photogrammetry (SCP), Laser altimeter^a^, *Manned aircraft*^b^ | Total length^1^, Body widths^2^, Body lengths^3^ | Morphometrics^A^, Body condition^B^, Demographics^C^, Development^D^, Comparative physiology^E^, Foraging dynamics^F^ | Kotik *et al.* (2023)^a,2,3,A,B,D,E^; Durban *et al.* (2021)^1,2,A,B,E^; Stewart *et al.* (2021*a*)^a,b,2,3,B,C,F^; Fearnbach *et al.* (2020)^1,2,3,B^; Groskreutz *et al.* (2019)^a,1,3,A,D^; Durban *et al.* (2015*a*)^1,A^ |
| *Globicephala macrorhynchus*  Short-finned pilot whale | Commercial multirotor | Photogrammetry (SCP), Laser altimeter | Total length, Body widths, Body lengths, Body volume | Morphometrics, Body condition, Demographic units, Comparative physiology | Arranz *et al.* (2022) |
| *Phocoena phocoena*  Harbour porpoise | Commercial multirotor | Photogrammetry (SCP), Laser altimeter, *Video cameras* | Total length, Body width | Morphometrics, Body condition | Irschick *et al.* (2021) |
| *Trichechus manatus*  Antillean manatee | Commercial multirotor & modified or custom multirotor | Photogrammetry (SCP), Laser altimeter | Total length, Body width, Body length | Morphometrics, Body condition | Ramos *et al.* (2022) |
| **Kinematics and Biomechanics** | | | | | |
| *Class: Chondrichthyes*, *Marine* | | | | | |
| *Mobula birostris*  Oceanic manta ray | Commercial multirotor | Photogrammetry (SCP), Video | Body length, Speed of movement, Behaviour, Location of anatomical landmarks | Fine-scale kinematics, Demographic units | Fong *et al.* (2022) |
| *Carcharhinus melanopterus*  Blacktip reef shark | Commercial multirotor | Photogrammetry (SCP), Video\ | Body length, Speed of movement, Behaviour, Location of anatomical landmarks | Fine-scale kinematics | Porter *et al.* (2020) |
| *Class: Reptilia*, *Marine* | | | | | |
| *Crocodylus acutus*  American crocodile | Commercial multirotor | Video | Behaviour, Location of anatomical landmarks, Footfall pattern | Fine-scale kinematics, Comparative physiology, Paleobiology | Farlow *et al.* (2018)^^^ |
| *Class: Mammalia* | | | | | |
| *Terrestrial: Inland* | | | | | |
| *Giraffa giraffa giraffa*  Giraffe | Commercial multirotor | Photogrammetry (SfM – 3D models), Video, *Laser rangefinder* | Body length, Speed of movement, Behaviour, Location of anatomical landmarks | Fine-scale kinematics | Basu *et al.* (2019) |
| *Marine* | | | | | |
| *Balaenoptera bonaerensis*  Antarctic minke whale | Commercial multirotor & modified or custom multirotor | Photogrammetry (SCP), Video^a^, Laser altimeter^b^, *Biologger*^c^, *Animal-borne cameras*^d^, *Sonar^e^* | Total length, Body widths^1^, Body lengths^2^, Surface area^3^, Behaviour^4^, Engulfment capacity^5^ | Morphometrics^A^, Foraging dynamics^B^, Comparative physiology^C^, Kinematic scaling^D^, Fine-scale kinematics^E^, Energy dynamics^F^ | Cade *et al.* (2023)^a,b,c,d,2,4,A,B,C,D,E^; Gough *et al.* (2022)^a,b,c,d,1,4,5,A,B,C,D,E,F^; Gough *et al.* (2021)^b,c,d,1,2,3,A,C,D,E,F^; Savoca *et al.* (2021)^b,c,d,e,1,5,A,B,C,F^; Kahane-Rapport *et al.* (2020)^a,b,c,d,1,3,5,B,C,D^; Gough *et al.* (2019)^b,c,d,1,A,C,D,E^ |
| *Balaenoptera musculus*  Blue whale | Commercial multirotor^*^, modified or custom multirotor^†^ | Photogrammetry (SCP), Video^a^, Laser altimeter^b^, *Biologger*^c^, *Animal-borne cameras*^d^, *Sonar*^e^ | Total length, Body widths^1^, Body lengths^2^, Surface area^3^, Behaviour^4^, Engulfment capacity^5^, Speed of movement^6^ | Morphometrics^A^, Foraging dynamics^B^, Comparative physiology^C^, Kinematic scaling^D^, Fine-scale kinematics^E^, Energy dynamics^F^ | Gough *et al*. (2022)^*,†,a,b,c,d,1,4,5,A,B,C,D,E,F^; Gough *et al*. (2021)^*,†,b,c,d,1,2,3,A,C,D,E,F^; Savoca *et al*. (2021)^*,†,b,c,d,e,1,5,A,B,C,F^; Kahane-Rapport *et al*. (2020)^*,†,a,b,c,d,1,3,5,B,C,D^; Torres *et al*. (2020)^*,a,e,1,4,6,B,E^; Gough *et al*. (2019)^*,†,b,c,d,1,A,C,D,E^ |
| *Balaenoptera edeni brydei*  Bryde’s whale | Commercial multirotor^*^, modified or custom multirotor^†^ | Photogrammetry (SCP), Video^a^, Laser altimeter^b^, *Biologger*^c^, *Animal-borne cameras*^d^, *Video cameras*^e^ | Total length, Body widths^1^, Body lengths^2^, Surface area^3^, Behaviour^4^, Distance travelled^5^ | Morphometrics^A^, Foraging dynamics^B^, Comparative physiology^C^, Kinematic scaling^D^, Fine-scale kinematics^E^, Energy dynamics^F^ | Izadi *et al.* (2022)^*,a,c,e,4,5,B,E,^^; Gough *et al*. (2021)^*,†,b,c,d,e,1,2,3,A,C,D,E,F^; Gough *et al*. (2019)^*,†,b,c,d,1,A,C,D,E^ |
| *Balaenoptera physalus*  Fin whale | Commercial multirotor & modified or custom multirotor | Photogrammetry (SCP), Video^a^, Laser altimeter^b^, *Biologger*^c^, *Animal-borne cameras*^d^, *Sonar*^e^ | Total length, Body widths^1^, Body lengths^2^, Surface area^3^, Behaviour^4^, Engulfment capacity^5^ | Morphometrics^A^, Foraging dynamics^B^, Comparative physiology^C^, Kinematic scaling^D^, Fine-scale kinematics^E^, Energy dynamics^F^ | Gough *et al*. (2022)^a,b,c,d,1,4,5,A,B,C,D,E,F^; Gough *et al*. (2021)^b,c,d,1,2,3,A,C,D,E,F^; Savoca *et al*. (2021)^b,c,d,e,1,5,A,B,C,F^; Kahane-Rapport *et al*. (2020)^a,b,c,d,1,3,5,B,C,D^; Gough *et al*. (2019)^b,c,d,1,A,C,D,E^ |
| *Balaenoptera borealis*  Sei whale | Commercial multirotor & modified or custom multirotor | Photogrammetry (SCP), Video Laser altimeter, *Biologger*, *Animal-borne cameras* | Total length, Body widths, Surface area | Morphometrics, Comparative physiology, Kinematic scaling, Fine-scale kinematics, Energy dynamics | Gough *et al.* (2021) |
| *Megaptera novaeangliae*  Humpback whale | Commercial multirotor^*^, modified or custom multirotor^†^ | Photogrammetry (SCP), Video^a^, Laser altimeter^b^, *Biologger*^c^, *Animal-borne cameras*^d^, *Sonar*^e^, *Video cameras*^f^ | Total length, Body widths^1^, Body lengths^2^, Surface area^3^, Behaviour^4^, Engulfment capacity^5^, Speed of movement^6^ | Morphometrics^A^, Foraging dynamics^B^, Comparative physiology^C^, Kinematic scaling^D^, Fine-scale kinematics^E^, Energy dynamics^F^ | Gough *et al*. (2022)^*,†,a,b,c,d,1,4,5,A,B,C,D,E,F^; Gough *et al*. (2021)^*,†,b,c,d,1,2,3,A,C,D,E,F^; Savoca *et al*. (2021)^*,†,b,c,d,e,1,5,A,B,C,F^; Kahane-Rapport *et al*. (2020)^*,†,a,b,c,d,1,3,5,B,C,D^; Gough *et al*. (2019)^*,†,b,c,d,1,A,C,D,E^; Werth *et al*. (2019)^*,a,f,1,2,4,6,A,B,E^^ |
| **Bioenergetics** | | | | | |
| *Class: Aves, Freshwater* | | | | | |
| *Cygnus olor*  Mute swan | Commercial multirotor | Video | Behaviour, Distance travelled | Impacts of external factors (disturbance), Energy dynamics | Clausen *et al.* (2020)^^^ |
| *Class: Mammalia* | | | | | |
| Terrestrial: Coastal | | | | | |
| *Ursus maritimus*  Polar bear | Commercial multirotor | Video | Behaviour | Foraging dynamics | Jagielski *et al.* (2021a)ˇ; Jagielski *et al*. (2021b)ˇ |
| *Halichoerus grypus*  Gray seal | Commercial multirotor | Photogrammetry (SfM – Orthomosaic, 3D model, DSM) | Total length, Body volume | Body condition, Development, Energy dynamics | Shero *et al.* (2021) |
| *Marine* | | | | | |
| *Eubalaena glacialis*  North Atlantic right whale | Modified or custom multirotor | Photogrammetry (SCP), Laser altimeter, *Manned aircraft* | Total length | Development, Reproductive metrics | Stewart *et al.* (2022) |
| *Eubalaena australis*  Southern right whale | Commercial multirotor^*^, modified or custom multirotor^†^ | Photogrammetry (SCP), Video^a^, Laser altimeter^b^, *Hydrophone*^c^ | Total length, Body widths^1^, Body lengths^2^, Body volume^3^, Respiration rate^4^, Behaviour^5^, Speed of movement^6^, Injury assessment^7^, Nursing rate^8^, Skin condition^9^ | Morphometrics^A^, Body condition^B^, Demographic units^C^, Reproductive metrics^D^, Development^E^, Energy dynamics^F^, Impacts of external factor (disturbance^G^, injury^H^, predation^I^) | Charlton *et al*. (2023)^*,1,3,7,9,B,D,H^; Christiansen *et al.* (2023)^*,a,b,1, 3.4,5,6,A,B,C,D^; Sprogis *et al*. (2023)^*,a,c,4,5,6,8,E,F,G^; Christiansen *et al.* (2022*a*)^*,b,1,3,B,C,E^; Christiansen *et al*. (2022*b*)^*,b,1,3,A,B,D,E^; Johnston *et al.* (2022)^†,b,1,2,A,B,C^; Azizeh *et al.* (2021)^*,a,4,5,6,7,8,E,F,H,I^; Nielsen *et al*. (2019)^*,a,b,1,4,5,6,8,B,D,E,F^; Christiansen *et al.* (2018)^†,b,1,2,3,B,D,E^ |
| *Eschrichtius robustus*  Gray whale | Commercial multirotor^*^, modified or custom multirotor^†^ | Photogrammetry (SCP), Video^a^, Laser altimeter^b^, Temperature compensated barometer^c^, *Manned aircraft*^d^ | Total length, Body widths^1^, Body lengths^2^, Surface area^3^, Behaviour^4^, Body volume^5^ | Body condition^A^, Demographic units^B^, Energy dynamics^C^, Stress^D^, Demographics^E^, Comparative physiology^F^ | Stewart *et al*. (2023)^†,c,d,1,A,E^; Torres *et al*. (2022)^*,a,b,1,2,4,A,F^; Christiansen *et al.* (2021)^*,a,b,1,2,4,5,A,B^; Lemos *et al.* (2020*a*)^*,a,1,2,3,4,A,B^ |
| *Balaenoptera bonaerensis*  Antarctic minke whale | Commercial multirotor & modified or custom multirotor | Photogrammetry (SCP), Video^a^, Laser altimeter^b^, *Biologger*^c^, *Animal-borne cameras*^d^, *Sonar*^e^ | Total length, Body widths^1^, Body lengths^2^, Surface area^3^, Behaviour^4^, Engulfment capacity^5^ | Morphometrics^A^, Foraging dynamics^B^, Comparative physiology^C^, Kinematic scaling^D^, Fine-scale kinematics^E^, Energy dynamics^F^ | Cade *et al.* (2023)^a,b,c,d,1,4,A,B,C,D,E^; Gough *et al*. (2022)^a,b,c,d,1,4,5,A,B,C,D,E,F^; Gough *et al*. (2021)^b,c,d,1,2,3,A,C,D,E,F^; Savoca *et al*. (2021)^b,c,d,e,1,5,A,B,C,F^; Kahane-Rapport *et al*. (2020)^a,b,c,d,1,3,5,B,C,D^; Gough *et al*. (2019)^b,c,d,1,A,C,D,E^ |
| *Balaena mysticetus*  Bowhead whale | Commercial multirotor | Photogrammetry (SCP), Laser altimeter | Body widths, Body lengths, Body volume | Body condition, Demographic units, Reproductive metrics, Development, Energy dynamics, Foraging dynamics | Christiansen *et al.* (2024) |
| *Balaenoptera musculus*  Blue whale | Commercial multirotor^*^, modified or custom multirotor^†^ | Photogrammetry (SCP), Video^a^, Laser altimeter^b^, *Biologger*^c^, *Animal-borne cameras*^d^, *Sonar*^e^ | Total length, Body widths^1^, Body lengths^2^, Surface area^3^, Behaviour^4^, Engulfment capacity^5^, Speed of movement^6^ | Morphometrics^A^, Foraging dynamics^B^, Comparative physiology^C^, Kinematic scaling^D^, Fine-scale kinematics^E^, Energy dynamics^F^ | Gough *et al*. (2022)^*,†,a,b,c,d,1,4,5,A,B,C,D,E,F^; Gough *et al*. (2021)^*,†,b,c,d,1,2,3,A,C,D,E,F^; Savoca *et al*. (2021)^*,†,b,c,d,e,1,5,A,B,C,F^; Kahane-Rapport *et al*. (2020)^*,†,a,b,c,d,1,3,5,B,C,D,E^; Torres *et al*. (2020)^*,a,e,1,4,6,B,E^; Gough *et al*. (2019)^*,†,b,c,d,1,A,C,D,E^ |
| *Balaenoptera edeni brydei*  Bryde’s whale | Commercial multirotor^*^, modified or custom multirotor^†^ | Photogrammetry (SCP), Video^a^, Laser altimeter^b^, *Video cameras*^c^, *Biologger*^d^, *Animal-borne cameras*^e^ | Total length, Body widths^1^, Body lengths^2^, Surface area^3^, Behaviour^4^, Distance travelled^5^ | Morphometrics^A^, Foraging dynamics^B^, Comparative physiology^C^, Kinematic scaling^D^, Fine-scale kinematics^E^, Energy dynamics^F^ | Izadi *et al.* (2022)^*,a,c,d,4,5,B,F^^; Gough *et al*. (2021)^*,†,b,d,e,1,2,3,A,C,D,E,F^; Gough *et al*. (2019)^*,†,b,c,d,1,A,C,D,E^ |
| *Balaenoptera physalus*  Fin whale | Commercial multirotor & modified or custom multirotor | Photogrammetry (SCP), Video^a^, Laser altimeter^b^, *Biologger*^c^, *Animal-borne cameras*^d^, *Sonar^e^* | Total length, Body widths^1^, Body lengths^2^, Surface area^3^, Behaviour^4^, Engulfment capacity^5^ | Morphometrics^A^, Foraging dynamics^B^, Comparative physiology^C^, Kinematic scaling^D^, Fine-scale kinematics^E^, Energy dynamics^F^ | Gough *et al*. (2022)^a,b,c,d,1,4,5,A,B,C,D,E,F^; Gough *et al*. (2021)^b,c,d,1,2,3,A,C,D,E,F^; Savoca *et al*. (2021)^b,c,d,e,1,5,A,B,C,F^; Kahane-Rapport *et al*. (2020)^a,b,c,d,1,3,5,B,C,D^; Gough *et al*. (2019)^b,c,d,1,A,C,D,E^ |
| *Balaenoptera borealis*  Sei whale | Commercial multirotor & modified or custom multirotor | Photogrammetry (SCP), Video, Laser altimeter, *Biologger*, *Animal-borne cameras* | Total length, Body widths, Surface area | Morphometrics, Comparative physiology, Kinematic scaling, Fine-scale kinematics | Gough *et al.* (2021) |
| *Megaptera novaeangliae*  Humpback whale | Commercial multirotor^*^, modified or custom multirotor^†^ | Photogrammetry (SCP), Video^a^, Laser altimeter^b^, *Biologger*^c^, *Animal-borne cameras*^d^, *Sonar*^e^, *Tissue sampling (Lipid concentration)*^f^ | Total length, Body widths^1^, Body lengths^2^, Body volume^3^, Surface area^4^, Behaviour^5^, Respiration rate^6^, Engulfment capacity^7^ | Morphometrics^A^, Body condition^B^, Demographic units^C^, Reproductive metrics^D^, Development^E^, Foraging dynamics^F^, Comparative physiology^G^, Kinematic scaling^H^, Energy dynamics^I^, Migratory costs^J^, Fine-scale kinematics^K^ | Russell *et al.* (2023*a*)^*b,1,2,3,B,C,J^; Russell *et al*. (2022)^*1,2,3,B,C,E,J^; Ejrnæs & Sprogis (2021)^*a,1,5,6,7,E,I^; Gough *et al*. (2022)^*,†,a,b,c,d,1,4,5,7,A,F,G,H,I,K^; Savoca *et al*. (2021)^*,†,b,c,e,1,7,A,F,G,I^; Aoki *et al.* (2021*a*)^*,c,f,1,2,4,B,F,I^; Gough *et al.* (2021)^*,†,b,c,d,1,2,4,A,G,H,I,K^; Christiansen *et al*. (2020*b*)^*,b,f,1,4,B,C,E,I^; Kahane-Rapport *et al.* (2020)^*,†,a,b,c,d,1,4,7,F,G,H^; Gough *et al.* (2019)^*,†,b,c,d,1,A,G,H,K^; Christiansen *et al.* (2016*a*)^*,1,4,B,C,D,E^ |
| *Feresa attenuata*  Pygmy killer whale | Commercial multirotor | Photogrammetry (SCP), Laser altimeter, Video, *Tissue sampling (Lipid concentration)* | Total length, Body widths, Body volume, Behaviour | Body condition, Energy dynamics, Impacts of external factor (disturbance) | Currie *et al.* (2021) |
| *Orcinus orca*  Killer whale | Modified or custom multirotor | Photogrammetry (SCP), Laser altimeter, *Manned aircraft* | Body widths, Body lengths | Body condition, Foraging dynamics, Demographics | Stewart *et al.* (2021*a*) |
| **Vital Signs: Respiration, Heart Rate, and Temperature** | | | | | |
| *Class: Osteichthyes, Freshwater* | | | | | |
| *Salmo salar*  Atlantic salmon | Commercial multirotor | Thermal, Photogrammetry (SfM – Orthomosaic, Thermal Orthomosaic), *Temperature loggers* | Environmental surface temperatures | Thermal habitat quality, Demographic units | Morgan & O’Sullivan (2023) |
| *Salvelinus* *fontinalis*  Brook trout | Commercial multirotor | Thermal, Photogrammetry (SfM – Orthomosaic, Thermal Orthomosaic), *Temperature loggers* | Environmental surface temperatures | Thermal habitat quality, Demographic units | Morgan & O’Sullivan (2023) |
| *Class: Reptilia, Terrestrial: Inland* | | | | | |
| *Zootoca vivipara*  Viviparous lizard | Commercial multirotor | Photogrammetry (Orthomosaic), *Temperature loggers* | Habitat composition | Thermal habitat quality | Rozen-Rechels *et al.* (2021) |
| *Mesalina bahaeldini* | Commerical fixed-wing | Multispectral, Photogrammetry (SfM – Orthomosaic, Multispectral Orthomosaic, DSM), *Temperature loggers* | Habitat composition | Body condition, Thermal habitat quality | Stark *et al.* (2022) |
|  |  |  |  |  |  |
| *Crocodylus niloticus*  Nile crocodile | Commercial multirotor | Thermal, Photogrammetry (SfM – Orthomosaic, Thermal Orthomosaic), *Weather stations* | Behaviour, Body surface temperature, Environmental temperatures | Thermal habitat quality, Demographic units | Viljoen *et al*. (2023*b*) |
| *Class: Mammalia* | | | | | |
| *Terrestrial: Inland* | | | | | |
| *Aloutta palliata*  Howler monkey | Modified or custom multirotor | Video, Thermal | Body surface temperature | Temperature | Kays *et al.* (2019)^^^ |
| *Atelles geoffroyi*  Spider monkey | Modified or custom multirotor | Video, Thermal | Body surface temperature | Temperature | Kays *et al.* (2019)^^^ |
| *Sus scrofa*  Wild boar | Commercial multirotor | Video, Thermal | Body surface temperature, Decomposition stage, Heat loss anomalies | Disease surveillance | Rietz *et al.* (2023)^^^ |
| *Potos flavus*  Kinkajou | Modified or custom multirotor | Video, Thermal | Body surface temperature | Temperature | Kays *et al.* (2019)^^^ |
| *Marine* | | | | | |
| *Eubalaena glacialis*  North Atlantic right whale | Modified or custom multirotor | Video, Laser altimeter, Thermal^a^, Photogrammetry (SCP)^b^, Blow- collection (*specific gravity*)^c^*, Tissue sampling (histology)*^d^ | Behaviour, Heat loss anomalies^1^, Respiratory cycle duration^2^, Relative blowhole area^2^, Blow samples^2^ | Thermal physiology^A^ , Respiratory physiology^B^, Comparative physiology^B^ | Lonati *et al.* (2022)^a,d,1,A,^ˇ; Martins *et al.* (2020)^b,c,2,B^ |
| *Eubalaena australis*  Southern right whale | Commercial multirotor | Photogrammetry (SCP), Video, Laser altimeter^a^, *Hydrophone*^b^ | Total length, Respiration rate, Behaviour, Speed of movement, Nursing rate, Injury assessment^1^, Body widths^2^ | Development, Energy dynamics, Body condition^A^, Reproductive metrics^B^, Impacts of external factor (disturbance^C^, injury^D^, predation^E^) | Sprogis *et al*. (2023)^b,,C,^; Azizeh *et al*. (2021)^1,D,E^; Nielsen *et al*. (2019)^a,A,B^ |
| *Megaptera novaeangliae*  Humpback whale | Commercial multirotor^*^, modified or custom multirotor^†^ | Video, Photogrammetry (SCP)^a^, Thermal^b^, Laser altimeter^c^, Blow-collection (specific gravity)^d^, *Wavelet & spectral analyses*^e^ | Behaviour, Respiration rate^1^, Total length^2^, Body widths^2^, Respiratory cycle duration^3^, Relative blowhole area^3^, Blow samples^3^, Body surface temperature^4^, Blow temperature^4^, Heat loss anomalies^4^ | Energy dynamics^A^, Development^A^, Respiratory physiology^B^, Comparative physiology^B^, Respiration rate^C^, Heart rate^C^, Temperature^C^ | Ejrnæs & Sprogis (2021)^*,a,1,2,A^; Martins *et al.* (2020)^†,a,c,d,1,3,d,B,^ˇ; Horton *et al.* (2019)^*,b,e1,4,C,^^ |
| *Lagenorhynchus obscurus*  Dusky dolphin | Commercial multirotor | Video | Behaviour, Respiration rate, Speed of movement, Nursing rate^1^ | Respiration rate, Demographic units, Reproductive metrics^A^, Development^B^ | Orbach *et al.* (2020)^A,^ˇ; Weir *et al.* (2018)^1,B,^ˇ |
| *Class: Not specified, Terrestrial: Coastal* | | | | | |
| Not specified | Not specified | Photogrammetry (SfM) – DSM | Habitat composition, Topography | Thermal habitat quality, Energy dynamics | Choi *et al.* (2019) |
| **Microbiome, Endocrinology, and Genetics** | | | | | |
| *Class: Mammalia*, *Marine* | | | | | |
| *Eschrichtius robustus*  Gray whale | Commercial multirotor | Photogrammetry (SCP), Video^a^, Laser altimeter^b^, *Faecal sampling (Endocrine)*^c^ | Total length, Body width, Body lengths, Surface area^1^, Behaviour^2^, Injury assessment^3^ | Body condition, Demographic units^A^, Reproductive metrics^B^, Energy dynamics^C^, Impacts of external factor (disturbance^D^, injury^E^), Stress^F^ | Fernandez Ajó *et al*. (2023)^c,A,B^; Pirotta *et al*. (2023)^b,c,1,A,D,F^; Lemos *et al*. (2022*a*)^a,b,c,2,D,F^; Lemos *et al*. (2022*b*)^c,1,C,F^; Lemos *et al*. (2020*b*)^c,2,3,A,B,E,F^ |
| *Balaenoptera bonaerensis*  Antarctic minke whale | Modified or custom multirotor | Photogrammetry (SCP), Laser altimeter, *Tissue sampling (Genetic*, *Endocrine)* | Total length, Body widths | Genetic profile, Demographic units, Reproductive metrics, Demographics | Pallin *et al.* (2022) |
| *Balaenoptera musculus*  Blue whale | Modified or custom multirotor | Blow-collection (*Microbiome*, *Genetic*, *Endocrine*^a^), Video^a^ | Blow samples, Behaviour^1^ | Microbiome composition, Genetic profile^A^, Demographic units^A^, Stress^A^, Reproductive metrics^A^, Disease surveillance^B^ | Atkinson *et al.* (2021)^a,1,A^; Acevedo-Whitehouse *et al.* (2010)^B^ |
| *Balaenoptera edeni brydei*  Bryde’s whale | Modified or custom multirotor | Blow-collection (*Microbiome*) | Blow samples | Microbiome composition, Disease surveillance | Acevedo-Whitehouse *et al.* (2010) |
| *Balaenoptera physalus*  Fin whale | Modified or custom multirotor | Blow-collection (*Microbiome*) | Blow samples | Microbiome composition, Disease surveillance | Acevedo-Whitehouse *et al.* (2010) |
| *Megaptera novaeangliae*  Humpback whale | Modified or custom multirotor | Blow-collection (*Microbiome*^a^, *Genetic*^b^, *Endocrine*^c^, *Virome*^d^), Photogrammetry (SCP)^e^, Video^f^, *Tissue sampling (Genetic*, *Endocrine)*^g^, Laser altimeter^h^ | Blow samples, Behaviour^1^, Total length^2^, Body widths^2^ | Microbiome composition^A^, Body condition^B^, Genetic profile^C^, Demographic units^D^, Stress^E^, Reproductive metrics^F^, Disease surveillance^G^, Virome composition^H^, Comparative physiology^I^ | Atkinson *et al.* (2021)^a,b,c,e,1,A,C,D,E,F^; Bierlich *et al.* (2022)^e,g,h,2,B,D^; Geoghegan *et al.* (2018)^d,H,^^; Apprill *et al.* (2017)^a,A,G,I^; Pirotta *et al.* (2017)^a,1,A,G^ |
| *Physeter macrocephalus*  Sperm whale | Modified or custom multirotor | Blow-collection (*Microbiome*) | Blow samples | Microbiome composition, Disease surveillance^1^ | Centelleghe *et al.* (2020); Acevedo-Whitehouse *et al.* (2010)^1^ |
| *Sousa sahulensis*  Australian humpback dolphin | Modified or custom multirotor | Blow-collection (*Genetic*) | Blow samples, Behaviour | Genetic profile | Raudino *et al.* (2019) |
| *Tursiops truncates*  Bottlenose dolphin | Modified or custom multirotor | Blow-collection (*Microbiome*, *Genetic*^a^) | Blow samples, Behaviour^1^ | Microbiome composition, Genetic profile^A^ | Centelleghe *et al.* (2020); Raudino *et al.* (2019)^a,1,A^ |
| *Orcinus orca*  Killer whale | Modified or custom multirotor | Blow-collection (*Microbiome*, *Genetic*, *Endocrine*), Video | Blow samples, Behaviour | Microbiome composition, Genetic profile, Demographic units, Stress, Reproductive metrics | Atkinson *et al.* (2021) |
| **Disease and Injury** | | | | | |
| *Class: Mammalia* | | | | | |
| *Terrestrial: Inland* | | | | | |
| *Macaca fascicularis*  Long-tailed macaque | Not specified | Photogrammetry (SfM – Orthomosaic), *Satellite telemetry*, *Blood sampling (Pathology)* | Habitat loss | Disease surveillance, Spatial epidemiology | Stark *et al.* (2019)ˇ |
| *Macaca nemestrina*  Pig-tailed macaque | Commercial fixed-wing | Photogrammetry (SfM – Orthomosaic, 3D model), *Satellite telemetry* | Habitat loss, Habitat composition | Disease surveillance, Spatial epidemiology | Fornace *et al.* (2014)ˇ |
| *Dama dama*  Fallow deer | Modified or custom fixed-wing | Aerial photography, *Tissue sampling (Pathology)* | Habitat composition, Host spatial distribution | Disease surveillance, Spatial epidemiology | Laguna *et al.* (2018)ˇ; Barasona *et al.* (2014)ˇ |
| *Cervus elaphus*  Red deer | Modified or custom fixed-wing | Aerial photography, *Tissue sampling (Pathology)* | Habitat composition, Host spatial distribution | Disease surveillance, Spatial epidemiology | Laguna *et al.* (2018)ˇ; Barasona *et al.* (2014)ˇ |
| *Sus scrofa*  Wild boar | Commercial multirotor^*^, modified or custom fixed-wing^†^ | Video^a^, Thermal^a^, Aerial photography^b^, *Tissue sampling (Pathology)*^b^ | Body surface temperature^1^, Decomposition stage^1^, Heat loss anomalies^1^, Habitat composition^2^, Host spatial distribution^2^ | Disease surveillance, Spatial epidemiology^A^ | Rietz *et al.* (2023)^*,a,1,^^; Laguna *et al*. (2018)^†,b,2,A^; Barasona *et al*. (2014)^†,b,2,A^ |
| *Bos taurus*  Domestic cow | Modified or custom fixed-wing | Aerial photography, *Tissue sampling (Pathology)* | Habitat composition, Host spatial distribution | Disease surveillance, Spatial epidemiology | Laguna *et al.* (2018)ˇ; Barasona *et al.* (2014)ˇ |
| *Terrestrial: Coastal* | | | | | |
| *Arctocephalus pusillus doriferus*  Australian fur seal | Commercial multirotor | Photogrammetry (SfM – Orthomosaic) | Injury assessment | Injury prevalence (entanglement), Demographic units | Claro *et al.* (2019)ˇ |
| *Halichoerus grypus*  Gray seal | Commercial multirotor & modified or custom multirotor | Photogrammetry (SfM – Orthomosaic) | Injury assessment, Spatial distribution | Injury prevalence (entanglement) | Martins *et al.* (2019)ˇ |
| *Marine* | | | | | |
| *Eubalaena glacialis*  North Atlantic right whale | Modified or custom multirotor | Photogrammetry (SCP), Laser altimeter, *Manned aircraft* | Total length, Body widths, Injury assessment | Body condition, Development, Impact of external factor (injury) | Stewart *et al.* (2021*b*) |
| *Eubalaena australis*  Southern right whale | Commercial multirotor | Photogrammetry (SCP), Video^a^ | Total length, Injury assessment, Body widths^1^, Body volume^1^, Skin condition^1^, Speed of movement^2^, Respiration rate^2^, Nursing rate^2^ | Impact of external factor (injury, predation ^A^), Body condition^B^, Reproductive metrics^C^, Energy dynamics^D^, Development^E^ | Charlton *et al*. (2023)^1,B,C^; Azizeh *et al.* (2021)^a,2,A,D,E^ |
| *Eschrichtius robustus*  Gray whale | Commercial multirotor | Photogrammetry (SCP), Video, *Faecal sampling (Endocrine)* | Total length, Body width, Body lengths, Behaviour, Injury assessment | Body condition, Demographic units, Reproductive metrics, Impacts of external factor (injury), Stress | Lemos *et al.* (2020*b*) |
| *Balaenoptera musculus*  Blue whale | Commercial multirotor | Video | Injury assessment | Injury prevalence (entanglement), Demographic units | Ramp *et al.* (2021)ˇ |
| *Balaenoptera physalus*  Fin whale | Commercial multirotor | Video | Injury assessment, Skin condition^1^ | Injury prevalence (entanglement, predation^A^), Disease surveillance^A^, Demographic units^B^ | Herr *et al.* (2023)^1,A,^ˇ; Ramp *et al.* (2021)^B,^ˇ |
| *Megaptera novaeangliae*  Humpback whale | Commercial multirotor | Photogrammetry (SCP)^a^, Video^b^, Laser altimeter^c^ | Skin condition^1^, Surface area^1^, Injury assessment^2^, Total length^3^, Body lengths^3^, Behaviour^3^ | Disease surveillance^A^, Injury prevalence (entanglement)^B^, Demographic units^C^, Morphometrics^D^, Foraging dynamics^E^ | Leslie *et al.* (2022)^a,1,A^; Ramp *et al.* (2021)^b,2,B,^ˇ; Stepanuk *et al*. (2021)^a,c,C,D,E^ |
| *Delphinapterus leucas*  Beluga whale | Commercial multirotor | Video | Injury assessment | Impact of external factor (Injury) | Ryan *et al*. (2022)ˇ |

**REFERENCES**

ACEVEDO-WHITEHOUSE, K., ROCHA-GOSSELIN, A. & GENDRON, D. (2010). A novel non-invasive tool for disease surveillance of free-ranging whales and its relevance to conservation programs. *Animal Conservation* **13**(2), 217–225.

ALLAN, B. M., IERODIACONOU, D., HOSKINS, A. J. & ARNOULD, J. P. Y. (2019). A rapid UAV method for assessing body condition in fur seals. *Drones* **3**(1), 24.

ALVARADO, D. C., ROBINSON, P. W., FRASSON, N. C., COSTA, D. P. & BELTRAN, R. S. (2020). Calibration of aerial photogrammetry to estimate elephant seal mass. *Marine Mammal Science* **36**(4), 1347–1355.

AOKI, K., ISOJUNNO, S., BELLOT, C., IWATA, T., KERSHAW, J., AKIYAMA, Y., MARTÍN LÓPEZ, L. M., RAMP, C., BIUW, M., SWIFT, R., WENSVEEN, P. J., POMEROY, P., NARAZAKI, T., HALL, A., SATO, K. & MILLER, P. J. O. (2021*a*). Aerial photogrammetry and tag-derived tissue density reveal patterns of lipid-store body condition of humpback whales on their feeding grounds. *Proceedings of the Royal Society B: Biological Sciences* **288**(1943), 20202307.

APPRILL, A., MILLER, C. A., MOORE, M. J., DURBAN, J. W., FEARNBACH, H. & BARRETT-LENNARD, L. G. (2017). Extensive core microbiome in drone-captured whale blow supports a framework for health monitoring. *mSystems* **2**(5), e00119-17.

Arranz, P., Christiansen, F., Glarou, M., Gero, S., Visser, F., Oudejans, M. G., Aguilar de Soto, N. & Sprogis, K. (2022). Body condition and allometry of free-ranging short-finned pilot whales in the North Atlantic. *Sustainability* **14**(22), 14787.

ATKINSON, S., ROGAN, A., BAKER, C. S., DAGDAG, R., REDLINGER, M., POLINSKI, J., URBAN, J., SREMBA, A., BRANSON, M. & MASHBURN, K. (2021). Genetic, endocrine, and microbiological assessments of blue, humpback and killer whale health using unoccupied aerial systems. *Wildlife Society Bulletin* **45**(4), 654–669.

AZIZEH, T. R., SPROGIS, K. R., SOLEY, R., NIELSEN, M. L., UHART, M. M., SIRONI, M., MARÓN, C. F., BEJDER, L., MADSEN, P. T. & CHRISTIANSEN, F. (2021). Acute and chronic behavioral effects of kelp gull micropredation on southern right whale mother-calf pairs off Península Valdés, Argentina. *Marine Ecology Progress Series* **668**, 133–148.

Barasona, J. A., Mulero-Pázmány, M., Acevedo, P., Negro, J. J., Torres, M. J., Gortázar, C. & Vicente, J. (2014). Unmanned aircraft systems for studying spatial abundance of ungulates: relevance to spatial epidemiology. *PloS One* **9**(12), e115608.

BARLOW, D. R., BIERLICH, K., OESTREICH, W. K., CHIANG, G., DURBAN, J. W., GOLDBOGEN, J. A., JOHNSTON, D. W., LESLIE, M. S., MOORE, M. & RYAN, J. P. (2023). Shaped by their environment: Variation in blue whale morphology across three productive coastal ecosystems. *Integrative Organismal Biology* **5**(1), obad039.

BASU, C. K., DEACON, F., HUTCHINSON, J. R. & WILSON, A. M. (2019). The running kinematics of free-roaming giraffes, measured using a low cost unmanned aerial vehicle (UAV). *PeerJ* **7**, e6312.

Bierlich, K. C., Hewitt, J., Bird, C. N., Schick, R. S., Friedlaender, A., Torres, L. G., Dale, J., Goldbogen, J., Read, A. J., Calambokidis, J. & Johnston, D. W. (2021*a*). Comparing uncertainty associated with 1-, 2-, and 3D aerial photogrammetry-based body condition measurements of baleen whales. **8**, 2296–7745.

BIERLICH, K.C., HEWITT, J., SCHICK, R. S., PALLIN, L., DALE, J., FRIEDLAENDER, A. S., CHRISTIANSEN, F., SPROGIS, K., DAWN, A. H. & BIRD, C. N. (2022). Seasonal gain in body condition of foraging humpback whales along the western Antarctic Peninsula. *Frontiers in Marine Science* **9**, 1036860.

Bierlich, K.C., Kane, A., Hildebrand, L., Bird, C.N., Fernandez Ajo, A., Stewart, J., Hewitt, J., Hildebrand, I., Sumich, J. & Torres, L. (2023*a*). Downsized: gray whales using an alternative foraging ground have smaller morphology. *Biology Letters* **19**(8), 20230043.

BURNETT, J. D., LEMOS, L., BARLOW, D., WING, M. G., CHANDLER, T. & TORRES, L. G. (2019). Estimating morphometric attributes of baleen whales with photogrammetry from small uass: A case study with blue and gray whales. *Marine Mammal Science* **35**(1), 108–139.

CADE, D. E., KAHANE-RAPPORT, S. R., GOUGH, W. T., BIERLICH, K., LINSKY, J. M., CALAMBOKIDIS, J., JOHNSTON, D. W., GOLDBOGEN, J. A. & FRIEDLAENDER, A. S. (2023). Minke whale feeding rate limitations suggest constraints on the minimum body size for engulfment filtration feeding. *Nature Ecology & Evolution* **7**(4), 535–546.

CASA (2022). Drone weight categories and requirements. Australian Government Civil Aviation Safety Authority, Online.

CENTELLEGHE, C., CARRARO, L., GONZALVO, J., ROSSO, M., ESPOSTI, E., GILI, C., BONATO, M., PEDROTTI, D., CARDAZZO, B. & POVINELLI, M. (2020). The use of unmanned aerial vehicles (UAVs) to sample the blow microbiome of small cetaceans. *PLoS One* **15**(7), e0235537.

CHARLTON, C., CHRISTIANSEN, F., WARD, R., MACKAY, A. I., ANDREWS-GOFF, V., ZERBINI, A. N., CHILDERHOUSE, S., GUGGENHEIMER, S., SHANNESSY, B. O. & BROWNELL JR, R. L. (2023). Evaluating short-to medium-term effects of implantable satellite tags on southern right whales *Eubalaena australis*. *Diseases of Aquatic Organisms* **155**, 125–140.

Cheney, B. J., Dale, J., Thompson, P. M. & Quick, N. J. (2022). Spy in the sky: a method to identify pregnant small cetaceans. *Remote Sensing in Ecology and Conservation* **8**(4), 492–505.

Chenoweth, E. M., Houston, J., Burek Huntington, K. & Straley, J. M. (2022). A virtual necropsy: applications of 3D scanning for marine mammal pathology and education. *Animals* **12**(4), 527.

CHOI, F., GOUHIER, T., LIMA, F., RILOV, G., SEABRA, R. & HELMUTH, B. (2019). Mapping physiology: Biophysical mechanisms define scales of climate change impacts. *Conservation Physiology* **7**(1), coz028.

Christiansen, F., Bejder, L., Burnell, S., Ward, R. & Charlton, C. (2022*a*). Estimating the cost of growth in southern right whales from drone photogrammetry data and long-term sighting histories. *Marine Ecology Progress Series* **687**, 173-194.

CHRISTIANSEN, F., DAWSON, S. M., DURBAN, J. W., FEARNBACH, H., MILLER, C. A., BEJDER, L., UHART, M., SIRONI, M., CORKERON, P. & RAYMENT, W. (2020*a*). Population comparison of right whale body condition reveals poor state of the North Atlantic right whale. *Marine Ecology Progress Series* **640**, 1–16.

CHRISTIANSEN, F., DUJON, A. M., SPROGIS, K. R., ARNOULD, J. P. Y. & BEJDER, L. (2016*a*). Noninvasive unmanned aerial vehicle provides estimates of the energetic cost of reproduction in humpback whales. *Ecosphere* **7**(10), e01468.

Christiansen, F., Rodríguez-González, F., Martínez-Aguilar, S., Urbán, J., Swartz, S., Warick, H., Vivier, F. & Bejder, L. (2021). Poor body condition associated with an unusual mortality event in gray whales. *Marine Ecology Progress Series* **658,** 237–252.

CHRISTIANSEN, F., SIRONI, M., MOORE, M. J., DI MARTINO, M., RICCIARDI, M., WARICK, H. A., IRSCHICK, D. J., GUTIERREZ, R. & UHART, M. M. (2019). Estimating body mass of free‐living whales using aerial photogrammetry and 3D volumetrics. *Methods in Ecology and Evolution* **10**(12), 2034–2044.

CHRISTIANSEN, F., SPROGIS, K. R., GROSS, J., CASTRILLON, J., WARICK, H. A., LEUNISSEN, E. & BENGTSON NASH, S. (2020*b*). Variation in outer blubber lipid concentration does not reflect morphological body condition in humpback whales. *Journal of Experimental Biology* **223**(8), jeb213769.

CHRISTIANSEN, F., SPROGIS, K. R., NIELSEN, M. L., GLAROU, M. & BEJDER, L. (2023). Energy expenditure of southern right whales varies with body size, reproductive state and activity level. *Journal of Experimental Biology*, **226**(4), jeb245137.

Christiansen, F., Tervo, O., Heide-Jorgensen, M. & Teilmann, J. (2024). Prey consumption of bowhead whales in West Greenland estimated from drone measurements of body size and condition. *Polar Biology* **47**(1), 17–39.

CHRISTIANSEN, F., UHART, M. M., BEJDER, L., CLAPHAM, P., IVASHCHENKO, Y., TORMOSOV, D., LEWIN, N. & SIRONI, M. (2022*b*). Fetal growth, birth size and energetic cost of gestation in southern right whales. *The Journal of Physiology* **600**(9), 2245–2266.

CHRISTIANSEN, F., VIVIER, F., CHARLTON, C., WARD, R., AMERSON, A., BURNELL, S. & BEJDER, L. (2018). Maternal body size and condition determine calf growth rates in southern right whales. *Marine Ecology Progress Series* **592**, 267–281.

Christie, A. I., Colefax, A. P. & Cagnazzi, D. (2021). Feasibility of using small UAVs to derive morphometric measurements of Australian snubfin (*Orcaella heinsohni*) and humpback (*Sousa sahulensis*) dolphins. *Remote Sensing* **14**, 21.

CLARO, F., FOSSI, M. C., IOAKEIMIDIS, C., BAINI, M., LUSHER, A. L., MC FEE, W., MCINTOSH, R. R., PELAMATTI, T., SORCE, M., GALGANI, F. & HARDESTY, B. D. (2019). Tools and constraints in monitoring interactions between marine litter and megafauna: Insights from case studies around the world. **141**, 147–160.

CLAUSEN, K. K., HOLM, T. E., PEDERSEN, C. L., JACOBSEN, E. M. & BREGNBALLE, T. (2020). Sharing waters: The impact of recreational kayaking on moulting mute swans (*Cygnus olor*). *Journal of Ornithology* **161**, 469–479.

COLEFAX, A. P., KELAHER, B. P., PAGENDAM, D. E. & BUTCHER, P. A. (2020). Assessing white shark (*Carcharodon carcharias*) behavior along coastal beaches for conservation-focused shark mitigation. *Frontiers in Marine Science* **7**, 268.

Currie, J. J., van Aswegen, M., Stack, S. H., West, K. L., Vivier, F. & Bejder, L. (2021). Rapid weight loss in free ranging pygmy killer whales (*Feresa attenuata*) and the implications for anthropogenic disturbance of odontocetes. *Scientific Reports* **11**(1), 8181.

DAWSON, S. M., BOWMAN, M. H., LEUNISSEN, E. & SIRGUEY, P. (2017). Inexpensive aerial photogrammetry for studies of whales and large marine animals. *Frontiers in Marine Science* **4**, 366.

DE KOCK, M. E., O’DONOVAN, D., KHAFAGA, T. & HEJCMANOVÁ, P. (2021). Zoometric data extraction from drone imagery: The Arabian oryx (*Oryx leucoryx*). *Environmental Conservation* **48**(4), 295–300.

DE OLIVEIRA, L. L., ANDRIOLO, A., CREMER, M. J. & ZERBINI, A. N. (2023). Aerial photogrammetry techniques using drones to estimate morphometric measurements and body condition in South American small cetaceans. *Marine Mammal Science* **39**(3), 811–829.

Dickson, T., Rayment, W. & Dawson, S. (2021). Drone photogrammetry allows refinement of acoustically derived length estimation for male sperm whales. *Marine Mammal Science* **37**(3), 1150–1158.

DURBAN, J. W., FEARNBACH, H., BARRETT-LENNARD, L., PERRYMAN, W. & LEROI, D. (2015*a*). Photogrammetry of killer whales using a small hexacopter launched at sea. *Journal of Unmanned Vehicle Systems* **3**(3), 131–135.

DURBAN, J. W., FEARNBACH, H., PAREDES, A., HICKMOTT, L. & LEROI, D. (2021). Size and body condition of sympatric killer whale ecotypes around the Antarctic Peninsula. *Marine Ecology Progress Series* **677**, 209–217.

DURBAN, J. W., MOORE, M. J., CHIANG, G., HICKMOTT, L. S., BOCCONCELLI, A., HOWES, G., BAHAMONDE, P. A., PERRYMAN, W. L. & LEROI, D. J. (2015*b*). Photogrammetry of blue whales with an unmanned hexacopter. *Marine Mammal Science* **32**(4), 1510–1515.

EJRNÆS, D. D. & SPROGIS, K. R. (2021). Ontogenetic changes in energy expenditure and resting behaviour of humpback whale mother–calf pairs examined using unmanned aerial vehicles. *Wildlife Research* **49**(1), 34–45.

EZAT, M. A., FRITSCH, C. J. & DOWNS, C. T. (2018). Use of an unmanned aerial vehicle (drone) to survey Nile crocodile populations: A case study at Lake Nyamithi, Ndumo game reserve, South Africa. *Biological Conservation* **223**, 76–81.

FARLOW, J. O., ROBINSON, N. J., TURNER, M. L., BLACK, J. & GATESY, S. M. (2018). Footfall pattern of a bottom-walking crocodile (*Crocodylus acutus*). *Palaios* **33**(9), 406–413.

FEARNBACH, H., DURBAN, J. W., BARRETT‐LENNARD, L. G., ELLIFRIT, D. K. & BALCOMB, K. C. (2020). Evaluating the power of photogrammetry for monitoring killer whale body condition. *Marine Mammal Science* **36**(1), 359–364.

FERNANDEZ AJÓ, A., PIROTTA, E., BIERLICH, K., HILDEBRAND, L., BIRD, C. N., HUNT, K. E., BUCK, C. L., NEW, L., DILLON, D. & TORRES, L. G. (2023). Assessment of a non-invasive approach to pregnancy diagnosis in gray whales through drone-based photogrammetry and faecal hormone analysis. *Royal Society Open Science* **10**(7), 230452.

FONG, V., HOFFMANN, S. L. & PATE, J. H. (2022). Using drones to assess volitional swimming kinematics of manta ray behaviors in the wild. *Drones* **6**(5), 111.

Fornace, K. M., Drakeley, C. J., William, T., Espino, F. & Cox, J. (2014). Mapping infectious disease landscapes: Unmanned aerial vehicles and epidemiology. **30**(11), 514–519.

FORTUNE, S. M., KOSKI, W. R., HIGDON, J. W., TRITES, A. W., BAUMGARTNER, M. F. & FERGUSON, S. H. (2017). Evidence of molting and the function of “rock-nosing” behavior in bowhead whales in the eastern Canadian arctic. *PLoS One* **12**(11), e0186156.

FUDALA, K. & BIALIK, R. J. (2020). Breeding colony dynamics of southern elephant seals at Patelnia point, King George island, Antarctica. *Remote Sensing* **12**(18), 2964.

GEOGHEGAN, J. L., PIROTTA, V., HARVEY, E., SMITH, A., BUCHMANN, J. P., OSTROWSKI, M., EDEN, J. S., HARCOURT, R. & HOLMES, E. C. (2018). Virological sampling of inaccessible wildlife with drones. *Viruses* **10**(6), 300.

Glarou, M., Gero, S., Frantzis, A., Brotons, J. M., Vivier, F., Alexiadou, P., Cerdà, M., Pirotta, E. & Christiansen, F. (2023). Estimating body mass of sperm whales from aerial photographs. *Marine Mammal Science* **39**(1), 251–273.

GOEBEL, M. E., PERRYMAN, W. L., HINKE, J. T., KRAUSE, D. J., HANN, N. A., GARDNER, S. & LEROI, D. J. (2015). A small unmanned aerial system for estimating abundance and size of Antarctic predators. *Polar Biology* **38**, 619–630.

GOUGH, W. T., CADE, D. E., CZAPANSKIY, M. F., POTVIN, J., FISH, F. E., KAHANE-RAPPORT, S. R., SAVOCA, M. S., BIERLICH, K. C., JOHNSTON, D. W. & FRIEDLAENDER, A. S. (2022). Fast and furious: Energetic tradeoffs and scaling of high-speed foraging in rorqual whales. *Integrative Organismal Biology* **4**(1), obac038.

GOUGH, W. T., SEGRE, P. S., BIERLICH, K., CADE, D. E., POTVIN, J., FISH, F. E., DALE, J., DI CLEMENTE, J., FRIEDLAENDER, A. S. & JOHNSTON, D. W. (2019). Scaling of swimming performance in baleen whales. *Journal of Experimental Biology* **222**(20), jeb204172.

GOUGH, W. T., SMITH, H. J., SAVOCA, M. S., CZAPANSKIY, M. F., FISH, F. E., POTVIN, J., BIERLICH, K. C., CADE, D. E., DI CLEMENTE, J., KENNEDY, J., SEGRE, P., STANWORTH, A., WEIR, C. & GOLDBOGEN, J. A. (2021). Scaling of oscillatory kinematics and froude efficiency in baleen whales. *Journal of Experimental Biology* **224**(13), jeb237586.

Gray, P. C., Bierlich, K. C., Mantell, S. A., Friedlaender, A. S., Goldbogen, J. A., Johnston, D. W. & Ye, H. (2019). Drones and convolutional neural networks facilitate automated and accurate cetacean species identification and photogrammetry. *Methods in Ecology and Evolution* **10**(9), 1490–1500

GROSKREUTZ, M. J., DURBAN, J. W., FEARNBACH, H., BARRETT-LENNARD, L. G., TOWERS, J. R. & FORD, J. K. (2019). Decadal changes in adult size of salmon-eating killer whales in the eastern North Pacific. *Endangered Species Research* **40**, 183–188.

HERR, H., VIQUERAT, S., NAUJOCKS, T., GREGORY, B., LEES, A. & DEVAS, F. (2023). Skin condition of fin whales at Antarctic feeding grounds reveals little evidence for anthropogenic impacts and high prevalence of cookiecutter shark bite lesions. *Marine Mammal Science* **39**(1), 299–310.

Hirtle, N. O., Stepanuk, J. E., Heywood, E. I., Christiansen, F. & Thorne, L. H. (2022). Integrating 3D models with morphometric measurements to improve volumetric estimates in marine mammals. *Methods in Ecology and Evolution* **13**(11)**,** 2479 – 2490.

HODGSON, J. C., HOLMAN, D., TERAUDS, A., KOH, L. P. & GOLDSWORTHY, S. D. (2020). Rapid condition monitoring of an endangered marine vertebrate using precise, non-invasive morphometrics. *Biological Conservation* **242**, 108402.

HORTON, T. W., HAUSER, N., CASSEL, S., KLAUS, K. F., FETTERMANN, T. & KEY, N. (2019). Doctor drone: Non-invasive measurement of humpback whale vital signs using unoccupied aerial system infrared thermography. *Frontiers in Marine Science* **6**, 466.

INFANTES, E., CARROLL, D., SILVA, W. T., HÄRKÖNEN, T., EDWARDS, S. V. & HARDING, K. C. (2022). An automated work-flow for pinniped surveys: A new tool for monitoring population dynamics. *Frontiers in Ecology and Evolution* **10**, 905309.

INMAN, V. L., KINGSFORD, R. T., CHASE, M. J. & LEGGETT, K. E. (2019). Drone-based effective counting and ageing of hippopotamus (*Hippopotamus amphibius*) in the Okavango Delta in Botswana. *PLoS One* **14**(12), e0219652.

INMAN, V. L. & LEGGETT, K. E. (2022). Hidden hippos: Using photogrammetry and multiple imputation to determine the age, sex, and body condition of an animal often partially submerged. *Drones* **6**(12), 409.

Irschick, D. J., Martin, J., Siebert, U., Kristensen, J. H., Madsen, P. T. & Christiansen, F. (2021). Creation of accurate 3D models of harbor porpoises (*Phocoena phocoena*) using 3D photogrammetry. *Marine Mammal Science* **37**(2), 482–491.

Izadi, S., de Soto, N., Constantine, R. & Johnson, M. (2022). Feeding tactics of resident Bryde's whales in New Zealand. *Marine Mammal Science* **38**(3), 1104–1117.

JAGIELSKI, P. M., DEY, C. J., GILCHRIST, H. G., RICHARDSON, E. S., LOVE, O. P., & SEMENIUK, C. A. (2021*a*). Polar bears are inefficient predators of seabird eggs. *Royal Society Open Science*, **8**(4), 210391.

JAGIELSKI, P. M., DEY, C. J., GILCHRIST, H. G., RICHARDSON, E. S. & SEMENIUK, C. A. (2021*b*). Polar bear foraging on common eider eggs: Estimating the energetic consequences of a climate-mediated behavioural shift. *Animal Behaviour* **171**, 63–75.

Jech, J. M., Johnson, J. J., Lutcavage, M., Vanderlaan, A. S., Rzhanov, Y. & LeRoi, D. (2020). Measurements of juvenile Atlantic bluefin tuna (*Thunnus thynnus*) size using an unmanned aerial system. *Journal of Unmanned Vehicle Systems* **8**(2), 140–160.

Johnston, D. R., Rayment, W. & Dawson, S. M. (2022). Morphometrics and body condition of southern right whales on the calving grounds at Port Ross, Auckland Islands. *Mammalian Biology* **102**(4), 1525–1536.

KAHANE-RAPPORT, S. R., SAVOCA, M. S., CADE, D. E., SEGRE, P. S., BIERLICH, K. C., CALAMBOKIDIS, J., DALE, J., FAHLBUSCH, J. A., FRIEDLAENDER, A. S., JOHNSTON, D. W., WERTH, A. J. & GOLDBOGEN, J. A. (2020). Lunge filter feeding biomechanics constrain rorqual foraging ecology across scale. *Journal of Experimental Biology* **223**(20), jeb224196.

Kays, R., Sheppard, J., Mclean, K., Welch, C., Paunescu, C., Wang, V., Kravit, G. & Crofoot, M. (2019). Hot monkey, cold reality: surveying rainforest canopy mammals using drone-mounted thermal infrared sensors. *International Journal of Remote Sensing* **40**(2), 407–419.

KOTIK, C., DURBAN, J. W., FEARNBACH, H. & BARRETT‐LENNARD, L. G. (2023). Morphometrics of mammal‐eating killer whales from drone photogrammetry, with comparison to sympatric fish‐eating killer whales in the eastern North Pacific. *Marine Mammal Science* **39**(1), 42–58.

KRAUSE, D. J., HINKE, J. T., PERRYMAN, W. L., GOEBEL, M. E. & LEROI, D. J. (2017). An accurate and adaptable photogrammetric approach for estimating the mass and body condition of pinnipeds using an unmanned aerial system. *PLoS One* **12**(11), e0187465.

Laguna, E., Barasona, J. A., Triguero-Ocaña, R., Mulero-Pázmány, M., Negro, J. J., Vicente, J. & Acevedo, P. (2018). The relevance of host overcrowding in wildlife epidemiology: A new spatially explicit aggregation index. *Ecological Indicators* **84**, 695–700.

Larsen, H. L., Møller-Lassesen, K., Enevoldsen, E. M. E., Madsen, S. B., Obsen, M. T., Povlsen, P., Bruhn, D., Pertoldi, C. & Pagh, S. (2023). Drone with mounted thermal infrared cameras for monitoring terrestrial mammals. *Drones* **7**(1), 680.

LEMOS, L. S., BURNETT, J. D., CHANDLER, T. E., SUMICH, J. L. & TORRES, L. G. (2020*a*). Intra‐ and inter‐annual variation in gray whale body condition on a foraging ground. *Ecosphere* **11**(4), e03094.

LEMOS, L. S., HAXEL, J. H., OLSEN, A., BURNETT, J. D., SMITH, A., CHANDLER, T. E., NIEUKIRK, S. L., LARSON, S. E., HUNT, K. E. & TORRES, L. G. (2022*a*). Effects of vessel traffic and ocean noise on gray whale stress hormones. *Scientific Reports* **12**(1), 18580.

LEMOS, L. S., OLSEN, A., SMITH, A., BURNETT, J. D., CHANDLER, T. E., LARSON, S., HUNT, K. E. & TORRES, L. G. (2022*b*). Stressed and slim or relaxed and chubby? A simultaneous assessment of gray whale body condition and hormone variability. *Marine Mammal Science* **38**(2), 801–811.

Lemos, L. S., Olsen, A., Smith, A., Chandler, T. E., Larson, S., Hunt, K. & Torres, L. G. (2020*b*). Assessment of fecal steroid and thyroid hormone metabolites in eastern North Pacific gray whales. *Conservation Physiology* **8**(1), coaa110.

LESLIE, M. S., KANT, L., PERKINS-TAYLOR, C., VAN BRESSEM, M.-F., MINTON, G., MACDONALD, D., CHRISTIANSEN, F., WILLSON, M. S., COLLINS, T. & BALDWIN, R. (2022). Remote and non-invasive quantification of ‘Tattoo Skin Disease-Like’ dermatopathy in endangered Arabian Sea humpback whales using drone photography. *Mammalian Biology* **102**(4), 1605–1617.

LESLIE, M. S., PERKINS-TAYLOR, C. M., DURBAN, J. W., MOORE, M. J., MILLER, C. A., CHANARAT, P., BAHAMONDE, P., CHIANG, G. & APPRILL, A. (2020). Body size data collected non-invasively from drone images indicate a morphologically distinct Chilean blue whale (*Balaenoptera musculus*) taxon. *Endangered Species Research* **43**, 291–304.

LONATI, G. L., ZITTERBART, D. P., MILLER, C. A., CORKERON, P., MURPHY, C. T. & MOORE, M. J. (2022). Investigating the thermal physiology of critically endangered North Atlantic right whales (*Eubalaena glacialis*) via aerial infrared thermography. *Endangered Species Research* **48**, 139–154.

Marín-Enríquez, E., Charruau, P. & Félix-Salazar, L. A. (2023). Discovery of a suburban wetland refuge for a depleted American crocodile (*Crocodylus acutus*) population in northwestern Mexico, using a commercial unmanned aerial vehicle. *Tropical Conservation Science* **16**, 19400829231209848.

MARTINS, M. C. I., MILLER, C., HAMILTON, P., ROBBINS, J., ZITTERBART, D. P. & MOORE, M. (2020). Respiration cycle duration and seawater flux through open blowholes of humpback (*Megaptera novaeangliae*) and North Atlantic right (*Eubalaena glacialis*) whales. *Marine Mammal Science* **36**(4), 1160–1179.

MARTINS, M. C. I., SETTE, L., JOSEPHSON, E., BOGOMOLNI, A., ROSE, K., SHARP, S. M., NIEMEYER, M. & MOORE, M. (2019). Unoccupied aerial system assessment of entanglement in northwest Atlantic gray seals (*Halichoerus grypus*). *Marine Mammal Science* **35**(4), 1613–1624.

MORGAN, A. M. & O'SULLIVAN, A. M. (2023). Cooler, bigger; warmer, smaller: Fine-scale thermal heterogeneity maps age class and species distribution in behaviourally thermoregulating salmonids. *River Research and Applications* **39**(2), 163–176.

NIELSEN, M. L., SPROGIS, K. R., BEJDER, L., MADSEN, P. T. & CHRISTIANSEN, F. (2019). Behavioural development in southern right whale calves. *Marine Ecology Progress Series* **629**, 219–234.

OLEKSYN, S., TOSETTO, L., RAOULT, V., & WILLIAMSON, J. E. (2020). Drone-based tracking of the fine-scale movement of a coastal stingray (*Bathytoshia brevicaudata*). *Remote Sensing* **13**(1), 40.

Orbach, D. N., Eaton, J., Fiori, L., Piwetz, S., Weir, J. S., Würsig, M. & Würsig, B. (2020). Mating patterns of dusky dolphins (*Lagenorhynchus obscurus*) explored using an unmanned aerial vehicle. *Marine Mammal Science* **36**(4) 1097–1110.

PALLIN, L., BIERLICH, K., DURBAN, J., FEARNBACH, H., SAVENKO, O., BAKER, C., BELL, E., DOUBLE, M., DE LA MARE, W. & GOLDBOGEN, J. (2022). Demography of an ice-obligate mysticete in a region of rapid environmental change. *Royal Society Open Science* **9**(11), 220724.

PIACENZA, S. E., PIACENZA, J. R., FALLER, K. J., ROBINSON, N. J. & SIEGFRIED, T. R. (2022). Design and fabrication of a stereo-video camera equipped unoccupied aerial vehicle for measuring sea turtles, sharks, and other marine fauna. *PLoS One* **17**(10), e0276382.

PIROTTA, E., FERNANDEZ AJÓ, A., BIERLICH, K., BIRD, C. N., BUCK, C. L., HAVER, S. M., HAXEL, J. H., HILDEBRAND, L., HUNT, K. E. & LEMOS, L. S. (2023). Assessing variation in faecal glucocorticoid concentrations in gray whales exposed to anthropogenic stressors. *Conservation Physiology* **11**(1), coad082.

PIROTTA, V., SMITH, A., OSTROWSKI, M., RUSSELL, D., JONSEN, I. D., GRECH, A. & HARCOURT, R. (2017). An economical custom-built drone for assessing whale health. *Frontiers in Marine Science* **4,** 425.

POMEROY, P., O'CONNOR, L. & DAVIES, P. (2015). Assessing use of and reaction to unmanned aerial systems in gray and harbor seals during breeding and molt in the UK. *Journal of Unmanned Vehicle Systems* **3**(3), 102–113.

PORTER, M. E., RUDDY, B. T. & KAJIURA, S. M. (2020). Volitional swimming kinematics of blacktip sharks, *Carcharhinus limbatus*, in the wild. *Drones* **4**(4), 78.

RAHMAN, D. A., HERLIANSYAH, R., SUBHAN, B., HUTASOIT, D., IMRON, M. A., KURNIAWAN, D. B., SRIYANTO, T., WIJAYANTO, R. D., FIKRIANSYAH, M. H. & SIREGAR, A. F. (2023). The first use of a photogrammetry drone to estimate population abundance and predict age structure of threatened Sumatran elephants. *Scientific Reports* **13**(1), 21311.

Ramos, E. A., Landeo-Yauri, S., Castelblanco-Martínez, N., Arreola, M. R., Quade, A. H. & Rieucau, G. (2022). Drone-based photogrammetry assessments of body size and body condition of Antillean manatees. *Mammalian Biology* **102**(3), 765–779.

RAMP, C., GASPARD, D., GAVRILCHUK, K., UNGER, M., SCHLEIMER, A., DELARUE, J., LANDRY, S. & SEARS, R. (2021). Up in the air: Drone images reveal underestimation of entanglement rates in large rorqual whales. *Endangered Species Research* **44**, 33–44.

RAOULT, V. & GASTON, T. F. (2018). Rapid biomass and size-frequency estimates of edible jellyfish populations using drones. *Fisheries Research* **207**, 160–164.

Ratsimbazafindranahaka, M., Razafimahatratra, E., Mathevet, R., Adam, O., Huetz, C., Charrier, I. & Saloma, A. (2021). Morphometric study of humpback whale mother-calf pairs in the Sainte Marie channel, Madagascar, using a simple drone-based photogrammetric method. *Western Indian Ocean Journal of Marine Science* **20**(2), 95–107.

RAUDINO, H. C., TYNE, J. A., SMITH, A., OTTEWELL, K., MCARTHUR, S., KOPPS, A. M., CHABANNE, D., HARCOURT, R. G., PIROTTA, V. & WAPLES, K. (2019). Challenges of collecting blow from small cetaceans. *Ecosphere* **10**(10), e02901.

LACKNER, T., VON HOERMANN, C., CONRATHS, F. J. & MÜLLER, J. (2023). Drone-based thermal imaging in the detection of wildlife carcasses and disease management. *Transboundary and Emerging Diseases* **2023,** 5517000.

ROZEN‐RECHELS, D., RUTSCHMANN, A., DUPOUÉ, A., BLAIMONT, P., CHAUVEAU, V., MILES, D. B., GUILLON, M., RICHARD, M., BADIANE, A. & MEYLAN, S. (2021). Interaction of hydric and thermal conditions drive geographic variation in thermoregulation in a widespread lizard. *Ecological Monographs* **91**(2), e01440.

RUSSELL, G., CAGNAZZI, D., COLEFAX, A., SPROGIS, K. R. & CHRISTIANSEN, F. (2023*a*). Cost of migration and migratory timing in Western Australian humpback whales. *Marine Mammal Science* **40**, e13074.

RUSSELL, G., CAGNAZZI, D., COLEFAX, A., SPROGIS, K. R. & CHRISTIANSEN, F. (2023*a*). Cost of migration and migratory timing in Western Australian humpback whales. *Marine Mammal Science* **40**, e13074.

RUSSELL, G., CHRISTIANSEN, F., COLEFAX, A., SPROGIS, K. R., & CAGNAZZI, D. (2023*b*). Comparisons of morphometrics and body condition between two breeding populations of Australian humpback whales. *Wildlife Research* **51**, WR23026.

RUSSELL, G., COLEFAX, A., CHRISTIANSEN, F., RUSSELL, G., FOWLER, Z. & CAGNAZZI, D. (2022). Body condition and migration timing of east Australian humpback whales. *Marine Ecology Progress Series* **692**, 169–183.

Ryan, K. P., Petersen, S. D., Ferguson, S. H., Breiter, C. J. C. & Watt, C. A. (2022). Photographic evidence of tagging impacts for two beluga whales from the Cumberland Sound and western Hudson Bay populations. *Arctic Science* **8**(4), 1362–1368.

Savoca, M. S., Czapanskiy, M. F., Kahane-Rapport, S. R., Gough, W. T., Fahlbusch, J. A., Bierlich, K. C., Segre, P. S., Di Clemente, J., Penry, G. S., Wiley, D. N. & Calambokidis, J. (2021). Baleen whale prey consumption based on high-resolution foraging measurements. *Nature* **599**(7883), 85–90.

Sawan, S., Mondal, T., Williams, A., Yadav, S. & Krishnamurthy, R. (2023). Hybrid drone-based survey of riverine habitat and crocodiles in complex landscapes. *International Journal of Environmental Science and Technology* **20**(12), 13571–13582.

SETYAWAN, E., ERDMANN, M. V., MAMBRASAR, R., HASAN, A. W., SIANIPAR, A. B., CONSTANTINE, R., STEVENSON, B. C. & JAINE, F. R. (2022*a*). Residency and use of an important nursery habitat, Raja Ampat’s Wayag lagoon, by juvenile reef manta rays (*Mobula alfredi*). *Frontiers in Marine Science* **9**, 815094.

SETYAWAN, E., STEVENSON, B. C., IZUAN, M., CONSTANTINE, R. & ERDMANN, M. V. (2022*b*). How big is that manta ray? A novel and non-invasive method for measuring reef manta rays using small drones. *Drones* **6**(3), 63.

SHERO, M. R., DALE, J., SEYMOUR, A. C., HAMMILL, M. O., MOSNIER, A., MONGRAIN, S. & JOHNSTON, D. W. (2021). Tracking wildlife energy dynamics with unoccupied aircraft systems and three‐dimensional photogrammetry. *Methods in Ecology and Evolution* **12**(12), 2458–2472.

SPROGIS, K. R., HOLMAN, D., ARRANZ, P. & CHRISTIANSEN, F. (2023). Effects of whale-watching activities on southern right whales in Encounter Bay, South Australia. *Marine Policy* **150**, 105525.

STARK, D. J., FORNACE, K. M., BROCK, P. M., ABIDIN, T. R., GILHOOLY, L., JALIUS, C., GOOSSENS, B., DRAKELEY, C. J. & SALGADO-LYNN, M. (2019). Long-tailed macaque response to deforestation in a *Plasmodium knowlesi*-endemic area. *EcoHealth* **16**(4), 638–646.

STARK, G., MA, L., ZENG, Z.-G., DU, W.-G. & LEVY, O. (2022). Rocks and vegetation cover improve body condition of desert lizards during both summer and winter. *Integrative and Comparative Biology* **62**(4), 1031–1041.

STEPANUK, J. E., HEYWOOD, E. I., LOPEZ, J. F., DIGIOVANNI JR, R. A. & THORNE, L. H. (2021). Age-specific behavior and habitat use in humpback whales: Implications for vessel strike. *Marine Ecology Progress Series* **663**, 209–222.

Stewart, J., Durban, J., Europe, H., Fearnbach, H., Hamilton, P., Knowlton, A., Lynn, M., Miller, C., Perryman, W., Tao, B. & Moore, M. (2022). Larger females have more calves: influence of maternal body length on fecundity in North Atlantic right whales. *Marine Ecology Progress Series* **689,** 179–189.

Stewart, J. D., Durban, J. W., Fearnbach, H., Barrett‐Lennard, L. G., Casler, P. K., Ward, E. J. & Dapp, D. R. (2021*a*). Survival of the fattest: Linking body condition to prey availability and survivorship of killer whales. *Ecosphere* **12**(8), e03660.

STEWART, J. D., DURBAN, J. W., KNOWLTON, A. R., LYNN, M. S., FEARNBACH, H., BARBARO, J., PERRYMAN, W. L., MILLER, C. A. & MOORE, M. J. (2021*b*). Decreasing body lengths in North Atlantic right whales. *Current Biology* **31**(14), 3174–3179.e3.

STEWART, J. D., JOYCE, T. W., DURBAN, J. W., CALAMBOKIDIS, J., FAUQUIER, D., FEARNBACH, H., GREBMEIER, J. M., LYNN, M., MANIZZA, M. & PERRYMAN, W. L. (2023). Boom-bust cycles in gray whales associated with dynamic and changing arctic conditions. *Science* **382**(6667), 207–211.

STOKES, H. J., MORTIMER, J. A., LALOË, J. O., HAYS, G. C. & ESTEBAN, N. (2023). Synergistic use of UAV surveys, satellite tracking data, and mark‐recapture to estimate abundance of elusive species. *Ecosphere* **14**(3), e4444.

TORRES, L. G., BARLOW, D. R., CHANDLER, T. E. & BURNETT, J. D. (2020). Insight into the kinematics of blue whale surface foraging through drone observations and prey data. *PeerJ* **8**, e8906.

TORRES, L. G., BIRD, C. N., RODRÍGUEZ-GONZÁLEZ, F., CHRISTIANSEN, F., BEJDER, L., LEMOS, L., URBAN R, J., SWARTZ, S., WILLOUGHBY, A., HEWITT, J. & BIERLICH, K. C. (2022). Range-wide comparison of gray whale body condition reveals contrasting sub-population health characteristics and vulnerability to environmental change. *Frontiers in Marine Science* **9,** 876258.

TUCKER, J. P., COLEFAX, A. P., SANTOS, I. R., KELAHER, B. P., PAGENDAM, D. E. & BUTCHER, P. A. (2021). White shark behaviour altered by stranded whale carcasses: Insights from drones and implications for beach management. *Ocean & Coastal Management* **200**, 105477.

UNSD (2022). United Nations geoscheme. *United Nations*. Vol. 07 November 2022. Available at: https://unstats.un.org/unsd/methodology/m49/.

VERMEULEN, E., THAVAR, T., GLAROU, M., GANSWINDT, A. & CHRISTIANSEN, F. (2023). Decadal decline in maternal body condition of a southern ocean capital breeder. *Scientific Reports* **13**(1), 3228.

VILJOEN, D., WEBB, E., MYBURGH, J., TRUTER, C. & MYBURGH, A. (2023*a*). Remote body condition scoring of Nile crocodiles (*Crocodylus niloticus*) using uncrewed aerial vehicle derived morphometrics. *Frontiers in Animal Science* **4**, 1225396.

VILJOEN, D. M., WEBB, E. C., MYBURGH, J. G., TRUTER, J. C., LANG, J. W. & MYBURGH, A. (2023*b*). Adaptive thermal responses of captive Nile crocodiles (*Crocodylus niloticus*) in South Africa. *Applied Animal Behaviour Science* **269**, 106098.

Vivier, F., Wells, R. S., Hill, M. C., Yano, K. M., Bradford, A. L., Leunissen, E. M., Pacini, A., Booth, C. G., Rocho‐Levine, J. & Currie, J. J. (2023). Quantifying the age structure of free‐ranging delphinid populations: Testing the accuracy of unoccupied aerial system photogrammetry. *Ecology and Evolution* **13**(6), e10082.

WEIR, J. S., FIORI, L., ORBACH, D. N., PIWETZ, S., PROTHEROE, C. & WÜRSIG, B. (2018). Dusky dolphin (*Lagenorhynchus obscurus*) mother-calf pairs: An aerial perspective. *Aquatic Mammals* **44**(6), 603–607.

WERTH, A. J., KOSMA, M. M., CHENOWETH, E. M. & STRALEY, J. M. (2019). New views of humpback whale flow dynamics and oral morphology during prey engulfment. *Marine Mammal Science* **35**(4), 1556–1578.

Whitehead, D. A., Ayres, K. A., Gayford, J. H., Ketchum, J. T., Galván-Magana, F. & Christiansen, F. (2022). Aerial photogrammetry of whale sharks (*Rhincodon typus*) in the Bay of La Paz, using an unoccupied aerial vehicle. *Marine Biology* **169**(7), 1–8.
